# Effects of Simulated Herbivory on the Thermal Resilience of Two Temperate Seagrass Species

**DOI:** 10.1002/ece3.74069

**Published:** 2026-08-02

**Authors:** Niclas Einert, Claire Butler, Amanda K. Pettersen, Scott Bennett

**Affiliations:** ^1^ Institute for Marine and Antarctic Studies University of Tasmania Hobart Tasmania Australia; ^2^ School of Life and Environmental Sciences The University of Sydney Sydney New South Wales Australia

**Keywords:** herbivory, *Heterozostera*, interspecific thermal resilience, leaf‐size, marine foundation species, *Posidonia*, temperature‐size interactions, thermal performance

## Abstract

As climate change accelerates, increasing ocean temperatures and altered herbivory pressure are reshaping temperate marine ecosystems dominated by macrophyte foundation species. Despite the importance of these processes, it remains unclear how herbivory affects the thermal performance of macrophytes. We simulated herbivory by clipping the leaf length of two temperate seagrass species, *Posidonia australis* and *Heterozostera tasmanica*, and grew them at temperatures extending below and above their current thermal range (6°C–32°C). We assessed the effects of reduced leaf size (i.e., simulated herbivory) on thermal performance by quantifying growth, photosynthetic rates, leaf nutrient content and pigment concentrations. Responses to clipping treatment and growing temperature were species‐specific. Highly clipped *P. australis* showed higher photosynthetic rates and nutrient concentrations at 32°C, relative to low‐clipped and control plants, while growth did not differ between clipping treatments, and survival was high across treatments. In *H. tasmanica* , clipping lowered optimal growth temperatures, and survival declined sharply at 32°C across treatments. Our results indicate that simulated herbivory can increase the thermal resilience of *P. australis* but decreases the performance of *H. tasmanica* under thermal stress. Increased herbivory pressure under climate change may therefore have positive implications for some seagrasses, highlighting the importance of considering species‐specific responses when predicting the future resilience of seagrass ecosystems.

## Graphical abstract

This study investigates how reduced leaf size via simulated herbivory influences the thermal performance of temperate seagrasses. Simulated herbivory enhanced physiological performance and resilience under elevated temperatures in *Posidonia australis*, but not in *Heterozostera tasmanica*. These findings highlight species‐specific responses and emphasise the importance of trait‐based approaches when predicting seagrass responses to climate warming.

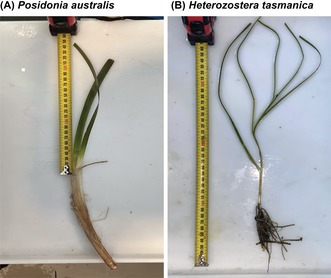

## 1 Introduction

Anthropogenic climate change is reshaping marine ecosystems globally, with pronounced effects on foundation species such as seagrasses, seaweeds and corals, which have experienced increased mortality and range contractions over the recent decades (Waycott et al. 2009; Wernberg et al. 2016; Hughes et al. 2018; Dunic et al. 2021). These declines arise from multiple direct and indirect impacts, among which ocean warming is widely regarded as a primary driver (Cooley et al. 2022). Globally, rising seawater temperatures can induce thermal stress, particularly for non‐mobile habitat‐forming species (Smale et al. 2019; Smith et al. 2024), alter nutrient availability (Hoegh‐Guldberg and Bruno 2010; Sun et al. 2024) and reconfigure species interactions (Bennett et al. 2015; Wernberg et al. 2016; Santana‐Garcon et al. 2023).

Warming waters are driving poleward shifts in species distributions and are increasing the occurrence of tropical and subtropical taxa in temperate waters (Last et al. 2011; Poloczanska et al. 2013) —a phenomenon known as tropicalisation (Vergés et al. 2014). In macrophyte‐dominated temperate ecosystems, this tropicalisation is associated with an increase in herbivory pressure from herbivorous fishes and urchins (Ling 2008; Wernberg et al. 2011). Herbivory reduces the above‐ground biomass (e.g., canopy height, shoot density) of macrophytes (Preen 1995; Scott et al. 2020), thereby reducing habitat complexity, with flow‐on effects for associated biodiversity (Smale et al. 2020; Jones et al. 2021). Consequently, increasing herbivory pressure is widely viewed as detrimental to the resilience of temperate marine ecosystems (Ling 2008; Vergés et al. 2014, 2016; Bennett et al. 2015; Martínez‐Crego et al. 2021), and is considered one of the biggest indirect effects of ocean warming (Duarte et al. 2018).

However, a recent translocation study observed unexpectedly high resilience to heat stress in cool‐edge seagrass populations of *Posidonia oceanica* following an intense episode of fish grazing (Bennett, Alcoverro, et al. 2022). In parallel, emerging evidence shows temperature‐driven reductions in the size of marine macrophytes, including seagrasses (Pansini et al. 2021; Agius et al. 2023; Marrone et al. 2025) and macroalgae (Alfonso et al. 2022; Wernberg et al. 2025). Together, these findings indicate potential benefits of reduced size under elevated ocean temperatures. Although Bennett, Alcoverro, et al. (2022) did not directly investigate the effects of reduced leaf size on thermal resilience, their observations raise the question of whether herbivory‐induced reductions in leaf size could similarly enhance thermal resilience relative to larger, ungrazed individuals. Heat‐stress responses in marine macrophytes are linked to a reallocation and investment of nutrients into the upregulation of several defence mechanisms (Henkel et al. 2009; Gu et al. 2012; Marín‐Guirao et al. 2016, 2017, 2018; Nguyen et al. 2021). Because increasing water temperatures can limit nutrient—particularly nitrogen—availability to macrophytes (Dean and Jacobsen 1986; Kwiatkowski et al. 2020), the resources that an individual can allocate to cellular heat protection are constrained. If the nutrient cost of heat defence scales with the amount of above‐ground tissue, plants with smaller canopies should incur lower costs and retain a larger share of available nutrients for thermal defence, potentially enhancing thermal resilience.

Another factor that modulates thermal resilience is the climatic conditions that a species has been exposed to over ecological and evolutionary time scales (Cameron et al. 2014; Kalambokidis and Travisano 2024). This eco‐evolutionary history can shape thermal performance through both phenotypic plasticity and genetic adaptation, to ultimately determine a species' potential to cope with increasing temperatures (Bennett, Vaquer‐Sunyer, et al. 2022). Comparing species with contrasting eco‐evolutionary histories can therefore help identify differences in thermal tolerance and vulnerability to future warming.

In this study, we hypothesise that reduced leaf size from herbivory increases the thermal resilience of marine macrophytes under heat stress. To test this hypothesis, we simulate herbivory and quantify its effects on the thermal performance of two temperate seagrasses from distinct evolutionary lineages, *Posidonia australis* (Posidoniaceae) and *Heterozostera tasmanica* (Zosteraceae) (Tuya et al. 2026). Although our primary aim is to understand how herbivory influences thermal performance, using two species allowed us to assess whether these effects are consistent across different seagrass species with contrasting growth forms and eco‐evolutionary histories. We experimentally manipulated leaf sizes via clipping to simulate varying herbivory intensities and characterised thermal performance curves for each species across clipping treatments. We then quantified how simulated herbivory altered thermal performance and associated physiological condition, using growth, survival, metabolic, nutrient and pigment responses as indicators of resilience. This study aims to advance our understanding of how the direct and indirect effects of ocean warming interact to influence the sensitivity of seagrasses under climate change. In doing so, this work supports more targeted forecasting and management of marine macrophyte ecosystems by identifying species and locations with heightened resilience or risk under future warming scenarios.

## 2 Material and Methods

### 2.1 Study Species and Sample Collection

**FIGURE 1:**
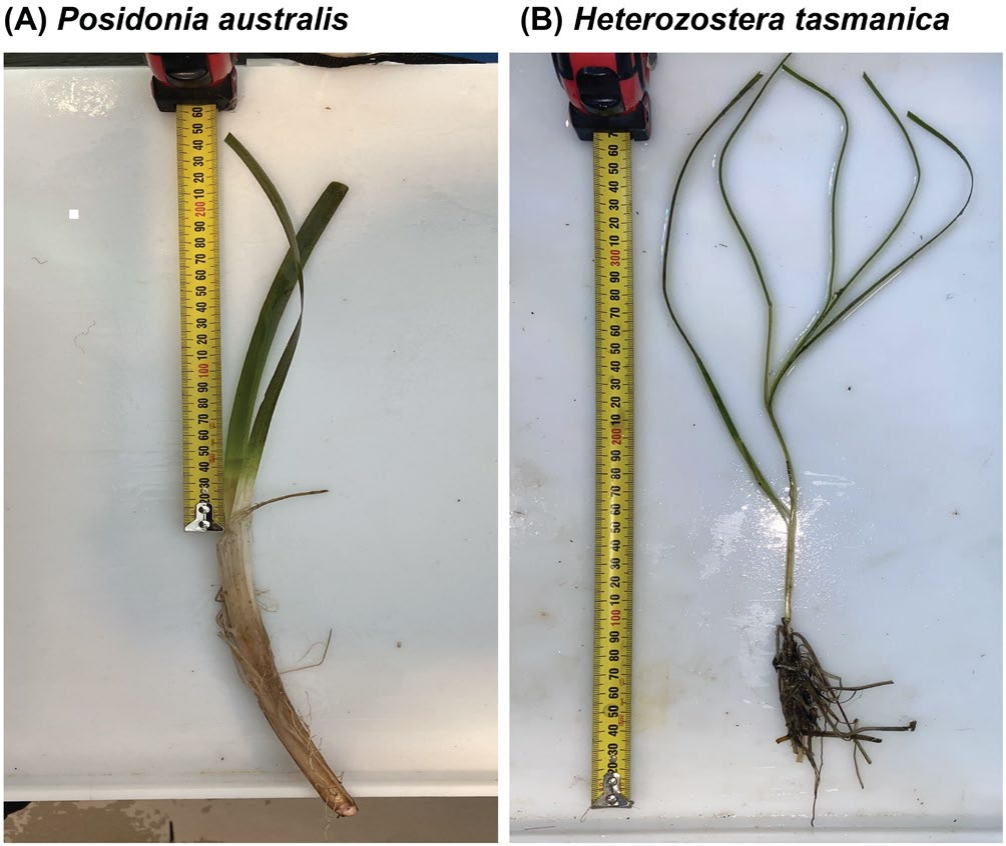
Photographs of study species *P. australis* (A) and *H. tasmanica* (B).

**FIGURE 2:**
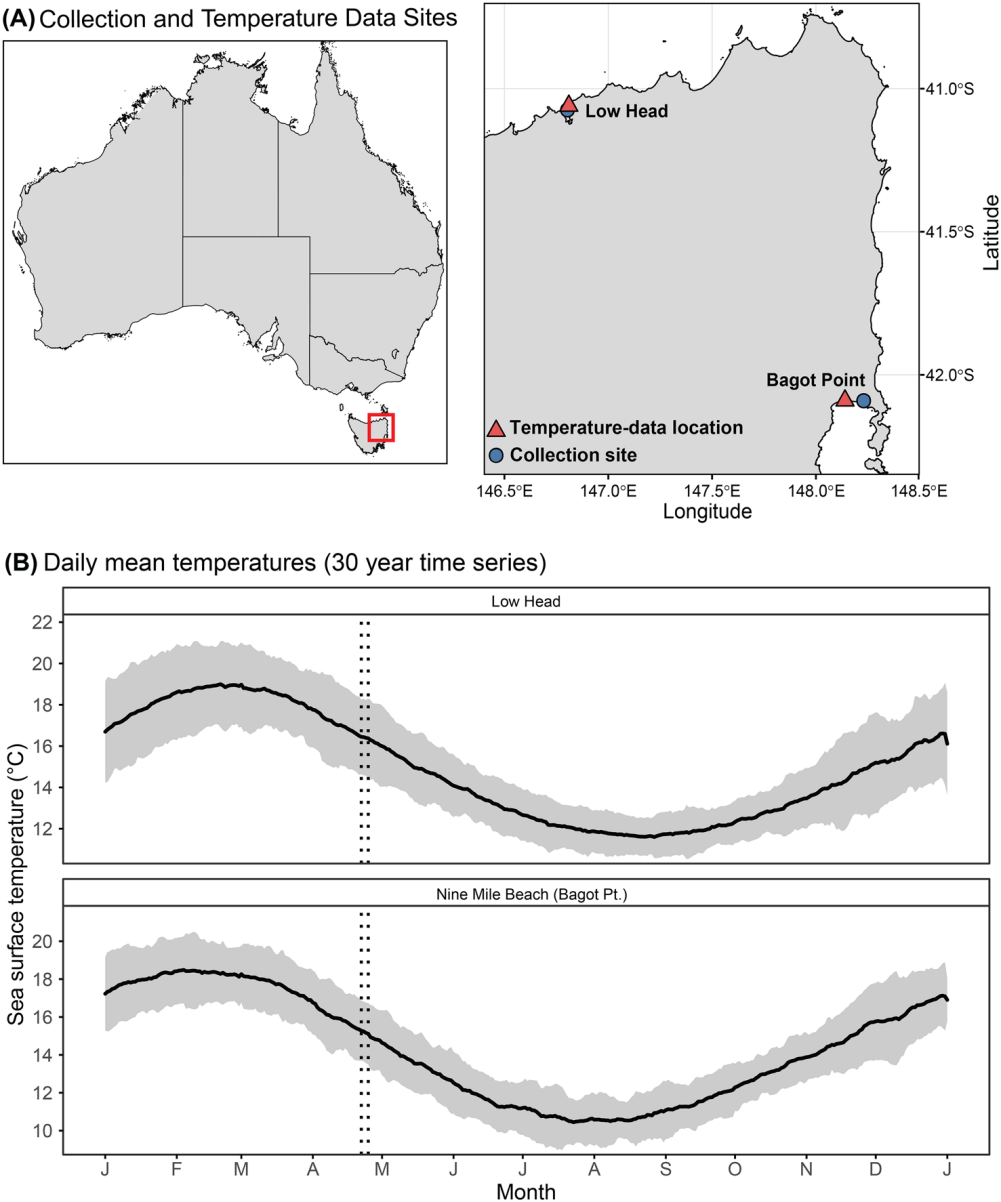
(A) Map of collection sites (blue circles) for *P. australis* (Low Head) and *H. tasmanica* (Bagot Point) and coordinates of points (red triangles) used to derive site‐specific temperature data (Ridgway and Ling 2023) in Tasmania, Australia; (B) Daily mean temperatures at the collection sites over a 30‐year time series (Ridgway and Ling 2023); Grey ribbon shows ±2 SD of mean (solid black line), dotted lines indicate time frame of sample collection.

The thermal performance experiment was carried out using two common temperate seagrass species in Australia, *Posidonia australis* J.D.Hooker and *Heterozostera tasmanica* (Ascherson) Hartog, collected from Low Head (Tamar River, Northern Tasmania) and Bagot Point (Swan River, Eastern Tasmania), respectively (Figures 1 and 2A). *P. australis* occurs on soft‐sediment substrates from just below low water to around 10 m depth and can form extensive subtidal meadows throughout southern coastline of Australia from Shark Bay in Western Australia to Northern New South Wales (Larkum and Den Hartog 1989; Creese et al. 2009; Aires et al. 2011). It has relatively long, broad leaves and large robust rhizomes used for the below‐ground storage of nutrients (Gobert et al. 2006). *H. tasmanica* has smaller, narrower leaves and shorter, thinner rhizomes than *P. australis* (compare Figure 1A,B) and occupies sheltered intertidal mudflats and shallow subtidal zones within temperate estuaries between South Australia, Victoria and north‐eastern Tasmania (Bulthuis and Woelkerling 1983; Kuo 2005). These species were selected to test whether the effects of simulated herbivory on thermal performance are consistent across seagrass growth forms or depend on species‐specific morphology and eco‐evolutionary history.

Seagrass shoots, including rhizomes, were collected between 22nd and 25th of April 2025 from 1 to 2 m depths at both locations. Collections were spaced out across each meadow (roughly 3 m between samples) to try and minimise relatedness between shoots (Bennett, Vaquer‐Sunyer, et al. 2022) and to reduce damage to the meadows. The collected shoots were put into damp calico bags and transported back to the laboratory within 4 h under dark and cool conditions (Bennett, Alcoverro, et al. 2022; Bennett, Vaquer‐Sunyer, et al. 2022). Upon arrival at the laboratory, samples were kept in aerated holding tanks at ambient seawater temperature (16°C) under a 12:12 light–dark cycle (200 μmol photons m^−2^ s^−1^) for three ( *H. tasmanica* ) to six ( *P. australis* ) days before the start of the experiment (laboratory acclimation period).

### 2.2 Experimental Design

The experiment was conducted in a temperature‐controlled room maintained at 16°C. Following laboratory acclimation, shoots were gently cleaned of epiphytes and grazing invertebrates. To standardise initial condition, leaves longer than 20 cm were clipped to a maximum length of 20 cm from meristem to leaf tip, while shorter, young leaves were left intact. Shoots without any leaves exceeding 20 cm were discarded. Rhizomes were standardised to the first internode for *P. australis* and to the fourth horizontal internode for *H. tasmanica* . Prepared shoots were transferred to individual experimental aquaria consisting of transparent plastic bags containing 2 L of filtered (1 μm), UV‐treated seawater (Savva et al. 2018; Bennett, Vaquer‐Sunyer, et al. 2022). Bags were suspended open‐topped (i.e., permitting gas exchange) within one of six 180 L temperature‐controlled water baths. Each bag contained an independent volume of seawater and separate plant material. Sediment was excluded to minimise the introduction of organisms and biological material that could affect water quality or confound treatment responses. Two shoots were placed in each bag for *H. tasmanica* at all temperatures and for *P. australis* at 26°C and 32°C to provide redundancy against mortality; otherwise, one shoot per bag was used (see Sections 2.3 and 2.4).

Following transfer, samples were acclimated for 48 h at 16°C, after which water‐bath temperatures were increased or decreased by 3°C every 24 h (Savva et al. 2018) until the assigned treatment temperature of either 6°C, 10°C, 16°C, 22°C, 26°C or 32°C was reached. These treatments extended beyond the temperatures naturally experienced by the source populations to characterise their full thermal responses (Figure 2B; Ridgway and Ling 2023). The 32°C treatment represented extreme heat stress comparable to temperatures occurring in shallow coastal habitats during severe marine heatwaves (Thomson et al. 2015; Neely et al. 2024).

Once treatment temperatures were reached, samples (*n* = 5 per species) were randomly assigned to one of three clipping treatments: Control (unaltered; longest leaf 20 cm), Low (longest leaf 16 cm; approximately 25% leaf‐biomass removal) or High (longest leaf 4 cm; approximately 80% removal), representing increasing intensities of simulated herbivory (Sanmartí et al. 2014; Garthwin et al. 2024). These treatments were not intended to reproduce herbivory levels at the collection sites, but rather to span moderate to intense levels of herbivory observed in similar seagrass systems around the world (Preen 1995; Kirsch et al. 2002; Prado et al. 2008; Planes et al. 2011). Clipping intensities were standardised across species to expose both to comparable levels of above‐ground biomass loss and enable a controlled assessment of species‐specific responses. Furthermore, clipping to 20 cm standardised the initial conditions and provided a procedural control in the ‘ungrazed’ samples. Samples were maintained at treatment temperature for 28 days.

**FIGURE 3:**
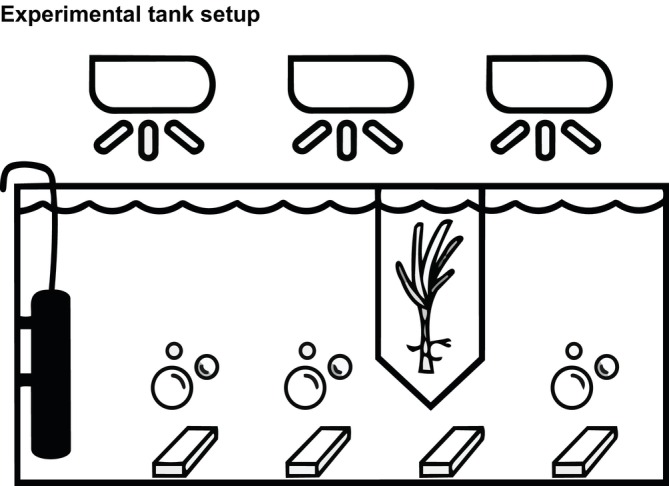
Schematic of experimental setup, showing one of six water baths (tanks), equipped with a heating/chilling unit, three full‐spectrum growth lights and porous Air Stones. Study species are kept in experimental aquaria (bags) suspended in the tank with their tops open for gas exchange.

Each 180 L tank contained a total of 30 bags and was fitted with a heating/chilling unit and continuously aerated to mix the water and maintain an even temperature distribution (Figure 3; Appendix 1). Full‐spectrum aquarium lamps (AquaZeal Malibu Z70, Shenzhen Ledzeal Green Lighting Co. Ltd.) provided a 12:12 h light–dark cycle. Light levels were measured before the experiment using a LI‐COR photometric bulb sensor and ranged from 185 to 300 μmol photons m^−2^ s^−1^. The light levels did not differ significantly among temperature treatments (ANOVA: *F*_5,42_ = 1.746, *p* = 0.145).

Tank temperatures were recorded throughout the experiment using Thermochron TC loggers (OnSolution Pty Ltd.) and remained within ±1.5°C of their target values. An exception occurred in the 22°C treatment, which reached 25°C for 2 days, following a power outage (Appendix 2). Seawater was sourced from Storm Bay, Tasmania and contained 28 μg L^−1^ ammonium, 330 μg L^−1^ nitrate + nitrite and 19 μg L^−1^ phosphate (Appendix 3). Water in each bag was replaced every 72 h to replenish dissolved nutrients and reduce the likelihood of nutrient limitation. Salinity was measured daily using a handheld WP‐81 multiprobe with a conductivity–temperature electrode (TPS Pty Ltd.) and maintained between 35 and 38 PSU, comparable to the collection sites, through water changes and the addition of Milli‐Q water where necessary.

### 2.3 Survival and Growth Measurements

To quantify survival, plants that had lost all leaves by the end of the experiment were classified as dead. For survivorship analyses, experimental bags were considered alive if at least one individual remained alive until the end of the experiment; bags were classified as dead only when all individuals had died.

The growth rate of each seagrass shoot was assessed by three different growth metrics. The leaf‐piercing technique as per Short and Duarte (2001) was used to obtain mass‐based growth (to nearest 1 mg) and length‐based growth (to the nearest mm). At the beginning of the 4‐week period, all leaves on each shoot were pierced with a hypodermic needle just above the meristem, and leaf growth was measured on all marked leaves as the distance from the needle mark to the meristem at the end of the experiment. For newly formed leaves that had emerged after marking, growth was measured as the distance from the meristem to the leaf tip. Leaf elongation rate was then calculated as total leaf elongation divided by the experimental duration (mm d^−1^). A growth conversion factor (g mm^−1^) was obtained by dividing the total leaf length by the wet weight of leaves (to the nearest 1 mg; Ohaus Scout STX, Ohaus Corporation) at the end of the growth experiment, and the mass‐based growth rate was calculated by multiplying this conversion factor with the total growth of the plant and dividing by the experimental duration (mg d^−1^). An additional relative growth rate was determined by standardising the mass‐based growth rate in mg d^−1^ over the total wet weight of the plant at the end of the experimental period (RGR d^−1^). For bags containing two individuals, growth rates were averaged when both individuals survived to the end of the experimental period. If one individual died, the growth rate of the surviving individual was taken as the bag's growth rate ( *P. australis* *n* = 4; *H. tasmanica* *n* = 4). If both individuals died, growth was treated as zero. Our primary objective was to compare the performance of different clipping treatments under temperature stress. The addition of extra shoots in high temperature treatments helped meet this objective and applying this method uniformly across all clipping treatments ensured comparable results between treatments.

### 2.4 Photosynthesis and Respiration Measurements

Net production (NP) of oxygen and plant respiration (R) were measured at the end of the growth period to obtain insights on the physiological performance of the study species (Britton et al. 2024; Busch et al. 2024). NP is the rate of O_2_ production under light conditions and R is the rate of O_2_ consumption, measured under dark conditions (Veenhof et al. 2024). Gross primary production (GPP) was estimated as the sum of NP and R (GPP = NP + R), assuming that R stayed the same under both light and dark conditions (Vaquer‐Sunyer et al. 2015; Bennett, Alcoverro, et al. 2022; Veenhof et al. 2024). A negative rate of NP (GPP < R) indicates a negative carbon balance and thus a lower capacity to sustain growth, indicating decreased physiological conditions (Lee et al. 2007; Collier et al. 2011; Marín‐Guirao et al. 2013).

Dissolved oxygen (DO) concentrations were measured using a Fibre Optic Oxygen Metre (PtSt 3 sensor spots and Fibox 4, PreSens GmbH; Silva et al. 2009). Shoots were placed in sealed glass jars (V = 1000 mL for *P. australis* , V = 300 mL for *H. tasmanica* ) and the change in DO was recorded over time (0.75 to 4 h depending on rates of change). We aimed to gain a change in DO of at least 5 to 10% based on previous observations in macrophyte measures (Butler et al. 2026). Jars were submerged in a water bath kept at the respective treatment temperature and placed on a shaker table to ensure adequate mixing. Net production measurements were carried out under full‐spectrum light at 200 μmol photons m^−2^ s^−1^. For respiration measurements, jars were wrapped in aluminium foil, and samples were left to acclimate over 30 min before the start of the measurements. For every treatment temperature and species, a plant‐free jar was measured concurrently as a control to account for any background oxygen production or consumption.

Absolute rates of GPP, NP and R were corrected according to background oxygen flux. Respiration rates smaller than the corresponding blank rate would have resulted in positive rates after correction and were set to zero instead (1 data point total). Respiration was standardised to whole‐plant wet weight (gWW^−1^), since the belowground organs (rhizome, roots, sheaths) of seagrasses contribute significantly to the plant's oxygen demand (Alcoverro et al. 2001). In *P. australis* , leaf weight was significantly lower than rhizome weight (pers. obs.); therefore, GPP and NP were expressed per unit leaf wet weight (gWWL^−1^) to account for potential effects of reduced leaf biomass. Where bags contained two individuals, we selected the individual in better physiological condition for the metabolic measurements and used the same individual for subsequent analyses (pigment content and C:N). This procedure was applied across all clipping treatments to ensure comparable results and the best chance of attaining measurements in high temperature treatments.

### 2.5 Pigment Content

At the end of the growth period, the concentrations of Chlorophyll *a* and *b* in the leaf tissue were measured to assess the composition of the photosynthetic apparatus (Anderson et al. 1995) and to support the interpretation of the observed photosynthetic rates (Croft et al. 2017). Leaf tissue samples were freeze‐dried, homogenised and approximately 5 mg of material was extracted with 100% ethanol overnight at 4°C (adapted from Nielsen et al. 2017). Extracted samples were centrifuged at 4°C and 4000 rpm and the absorption of the supernatant was measured spectrophotometrically at 649, 665 and 750 nm (HALO RB‐10, Dynamica Ltd.). Chlorophyll *a* and *b* concentrations were calculated using the following equations derived from the literature (Jespersen and Christoffersen 1987; Rowan 1989; Nielsen et al. 2017):

$$\begin{array}{c} {\mathrm{Chl}}_{a}=\frac{A_{665}-A_{750}*v*10^{3}}{77.9*M}\\ {\mathrm{Chl}}_{b}=\frac{A_{649}-A_{750}*v*10^{3}}{77.9*M} \end{array}$$

A_649,665,750_: absorption coefficients at 649, 665 and 750 nm, respectively.

v: Volume of ethanol used for the extraction.

M: Mass of dried leaf material used for the extraction.

### 2.6 Leaf Carbon and Nitrogen

Leaf carbon, nitrogen and C:N ratios were obtained at the end of the experiment as an indication of the physiological state of the samples (Britton et al. 2016). The remaining leaf tissue of each sample was dried at 60°C for 72 h and homogenously powdered (Jiang et al. 2023). Approximately 1.5 mg of homogenised sample was weighed into tin capsules, and tissue carbon and nitrogen content (% dry weight) were determined using an elemental analyser (FlashEA 1112, Thermo Scientific) at the Central Science Laboratory (University of Tasmania, Hobart). Results were calibrated using a certified sulphanilamide standard.

### 2.7 Statistical Analysis

All statistical analyses were performed in R, version 4.4.1 (R core team). Thermal performance curves (TPCs) were fitted to the measured growth and metabolic rates (GPP and NP) for each species and clipping treatment using the *rTPC* package (Padfield et al. 2021). A range of 8 to 10 thermal performance models was pre‐selected based on models commonly used for seagrass data (Adams et al. 2017). The best fit model for each dataset and species was chosen based on the criteria of Britton et al. (2024), consisting of (a) sensible shape of curve (unimodal with a clear peak, based on common thermal performance patterns), (b) ΔAIC‐score of < 2 relative to the best‐fit model with a sensible shape selected from criterion (a), and (c) plausibility of biologically relevant parameters (optimal temperature and maximum rate) and predicted confidence intervals of TPCs (Appendices 4 and 5). The optimal temperatures (*Topt*) and maximum rates (*Rmax*) and their bootsrapped 95% confidence intervals for each parameter were derived from models using the package *car* (Fox and Weisberg 2019). Relative growth rates of *P. australis* were several orders of magnitude smaller than 1 and to increase numerical stability these were multiplied by a constant factor of 1000. This affected the amplitude of the response parameter, but not the shape of curves, such that treatments could still be compared and that it allowed for interpretations of biological optima. After fitting, *Rmax* was back transformed to original units.

Distributions were cleaned by removing missing values (NAs), trimming 2% from each tail to reduce outlier influence and, for *Topt* only, excluding values within 0.5°C of experimental bounds (6°C and 32°C) to avoid boundary artefacts. Treatment effects were tested by using percentile‐bootstrap pairwise contrasts. Differences between each comparison (Control‐Low, Control‐High, Low‐High) were calculated by subtracting random draws from the bootstrapped distributions (100,000 draws with replacement) to create a new difference distribution. From this resulting difference distribution, 95% confidence intervals and two‐sided *p*‐values were derived, and *p*‐values were adjusted using Holm's procedure (*α* = 0.05).

For pigment and leaf nutrient (C:N) data, Generalised Additive Models (GAMs) were used to test for differences between clipping treatments, using the *mgcv* package (Wood 2011). GAMs were also used in cases where the data was too variable and no TPC could be fit (NP data of *P. australis* and GPP data of both species). Respiration data showed a near linear response to temperature such that a TPC could not be fit, so a linear mixed effects model was used for analysis (*lme4* package; Bates et al. 2015). All mixed‐effects models contained bag as a random effect to account for the potential influence of bag position in the tanks. This effect was never significant, so it was dropped in the subsequent analysis. Model diagnostics were examined and response parameters were transformed where assumptions such as homogeneity and normality of residuals were violated. If clipping treatment demonstrated a significant effect (*p* < 0.05) on a given response parameter, post hoc pairwise contrasts were estimated with *emmeans* (Lenth et al. 2025) using Tukey's HSD adjustment.

## 3 Results

### 3.1 Growth Rates and Survival

**FIGURE 4:**
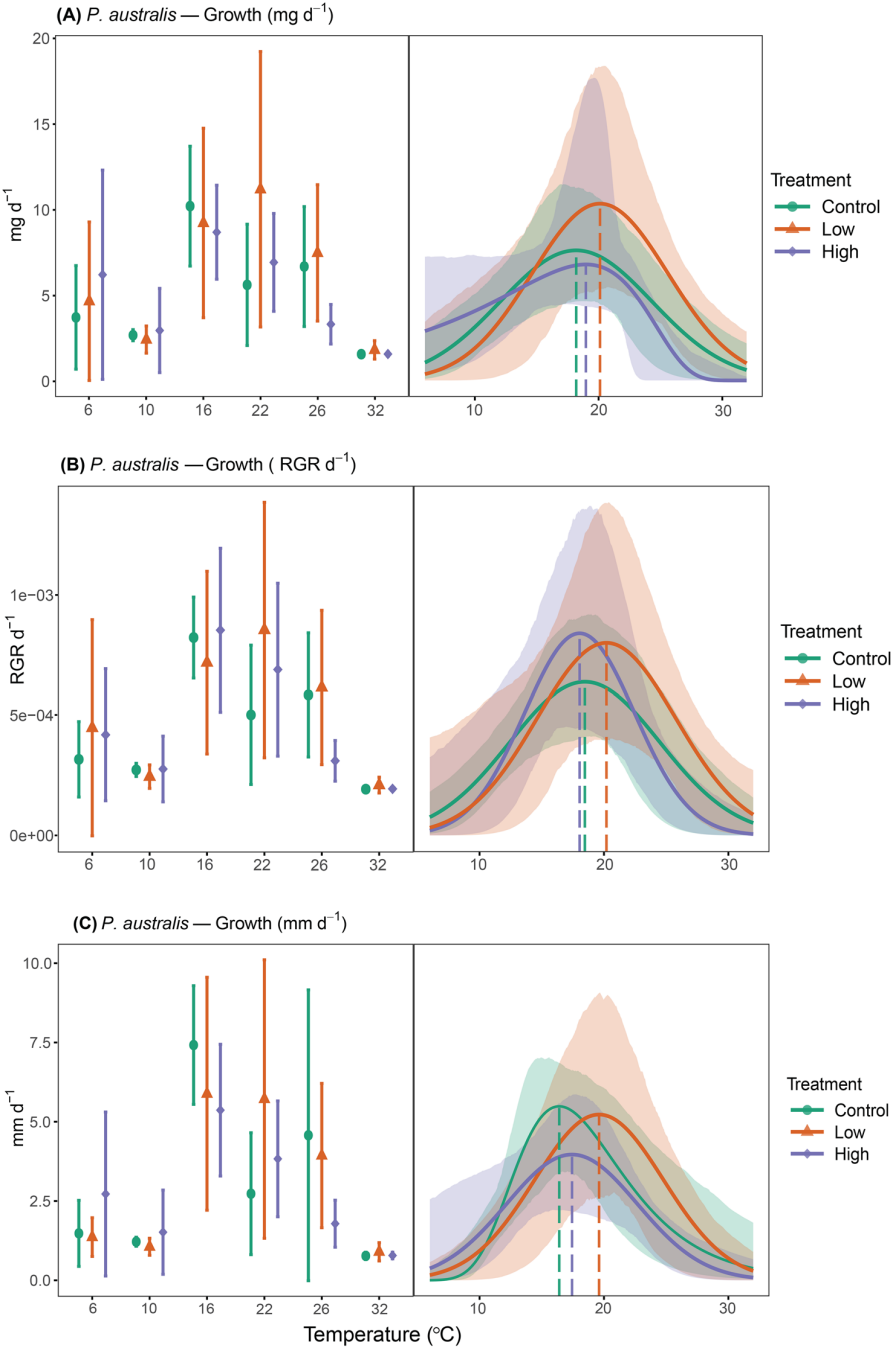
Growth responses and fitted thermal performance curves for the three growth measures of *P. australis* (A–C). Left panels show treatment means ± SD at each treatment temperature. Right panels show the corresponding thermal performance curves fitted. Lines represent model‐predicted means, shaded areas indicate 95% confidence intervals, and dashed lines intersect the curves at the estimated optimal temperatures (*Topt*).

**FIGURE 5:**
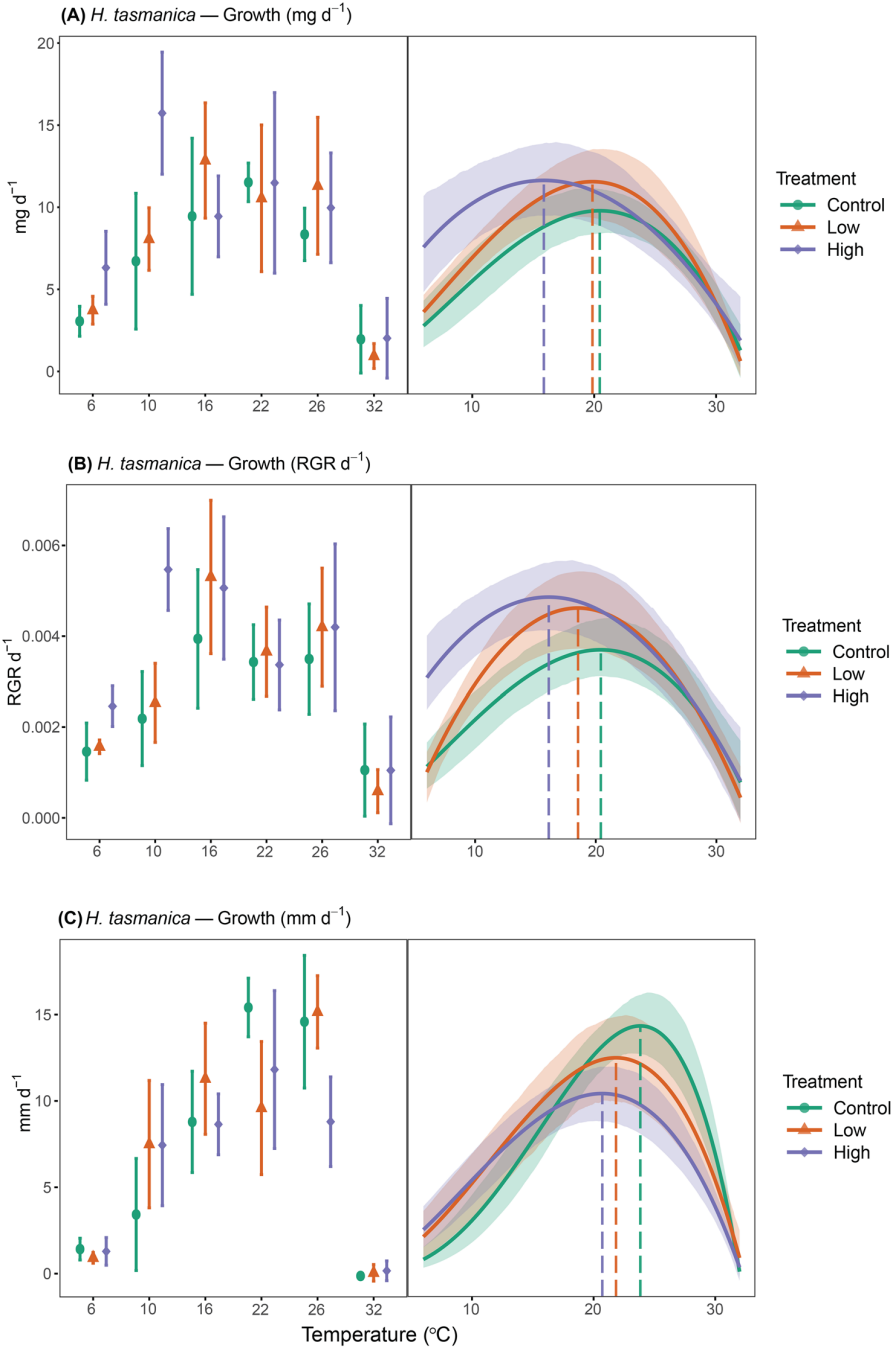
Growth responses and fitted thermal performance curves for the three growth measures of *H. tasmanica* (A–C). Left panels show treatment means ± SD at each treatment temperature. Right panels show the corresponding thermal performance curves fitted. Lines represent model‐predicted means, shaded areas indicate 95% confidence intervals, and dashed lines intersect the curves at the estimated optimal temperatures (*Topt*).

Growth rate showed a unimodal response to temperature for both *P. australis* and *H. tasmanica* across each of the three growth metrics (biomass‐based, length‐based and relative growth rates; Figures 4 and 5). For *P. australis* , growth rates and estimated optimal temperatures (*Topt*) did not differ significantly between clipping treatments (Table 1; Appendix 6). *Topt* ranged from 16.4°C to 20.2°C for the three growth responses. Survival of *P. australis* was high (80%–100%) across all clipping and temperature treatments.

**TABLE 1:** Estimated optimum temperatures (*Topt*) for growth response parameters with 95% confidence intervals (CIs) and estimated maximum rates of response parameters (*Rmax*) with 95% CIs; different lowercase letters indicate statistically significant differences among treatment groups (CT, control treatment; LT, low clipping treatment; HT, high clipping treatment).

Species	Growth measure	Treatment	*Topt* in °C (95% CI)	*Rmax* (95% CI)
*P. australis*	mg d^−1^	CT	18.2 (16.1–20.7)	7.95 (4.98–11.5)
		LT	20.1 (17.3–23.3)	10.3 (5.79–17.8)
		HT	19.0 (6.4–20.9)	6.76 (4.76–20.1)
	RGR d^−1^	CT	18.5 (16.5–20.7)	6.44e^−4^ (4.33e^−4^–9.33e^−4^)
		LT	20.2 (13.4–22.8)	8.01e^−4^ (4.47e^−4^–1.39e^−3^)
		HT	18.0 (15.7–19.9)	8.40e^−4^ (4.79e^−4^–1.43e^−3^)
	mm d^−1^	CT	16.4 (14.9–21.2)	5.49 (3.73–7.38)
		LT	19.6 (17.4–22.5)	5.23 (3.09–8.91)
		HT	17.4 (13.0–19.0)	3.97 (2.27–5.82)
*H. tasmanica*	mg d^−1^	CT	20.5 (18.3–22.2)^a^	9.79 (8.43–11.2)
		LT	19.9 (18.3–21.4)^a^	11.6 (9.44–13.8)
		HT	15.9 (12.0–18.7)^b^	11.6 (9.71–14.0)
	RGR d^−1^	CT	20.4 (18.6–22.5)^a^	3.70e^−3^ (3.11e^−3^–4.44e^−3^)^a^
		LT	18.5 (17.9–19.5)^b^	4.62e^−3^ (3.81e^−3^–5.44e^−3^)^ab^
		HT	16.1 (13.1–18.4)^c^	4.86e^−3^ (4.13e^−3^–5.76e^−3^)^b^
	mm d^−1^	CT	23.8 (22.6–24.9)^a^	14.3 (12.8–16.4)^a^
		LT	21.8 (19.8–23.7)^ab^	12.5 (10.3–15)^ab^
		HT	20.7 (18.9–22.3)^b^	10.4 (8.86–12.2)^b^

The growth of *H. tasmanica* was significantly influenced by clipping (Table 1; Appendix 6). Estimated optimum temperatures (*Topt*) of highly clipped plants were significantly lower than for control plants across all three growth metrics (*p* < 0.01 for all contrasts; Table 1). *Topt* ranged from 20.4°C to 23.8°C (control), 18.5°C to 21.8°C (low clipping) and 15.9°C to 20.7°C (high clipping). Additionally, estimated maximum growth rates (*Rmax*) of highly clipped plants were significantly lower than growth rates of control plants (*p* < 0.01). Survival did not differ between clipping treatments and was high across treatment temperatures with 100% survival at 6°C to 26°C. However, at 32°C, an abrupt decline of survival was detected, with only 40% survival across all three clipping treatments.

### 3.2 Photosynthesis and Respiration

**FIGURE 6:**
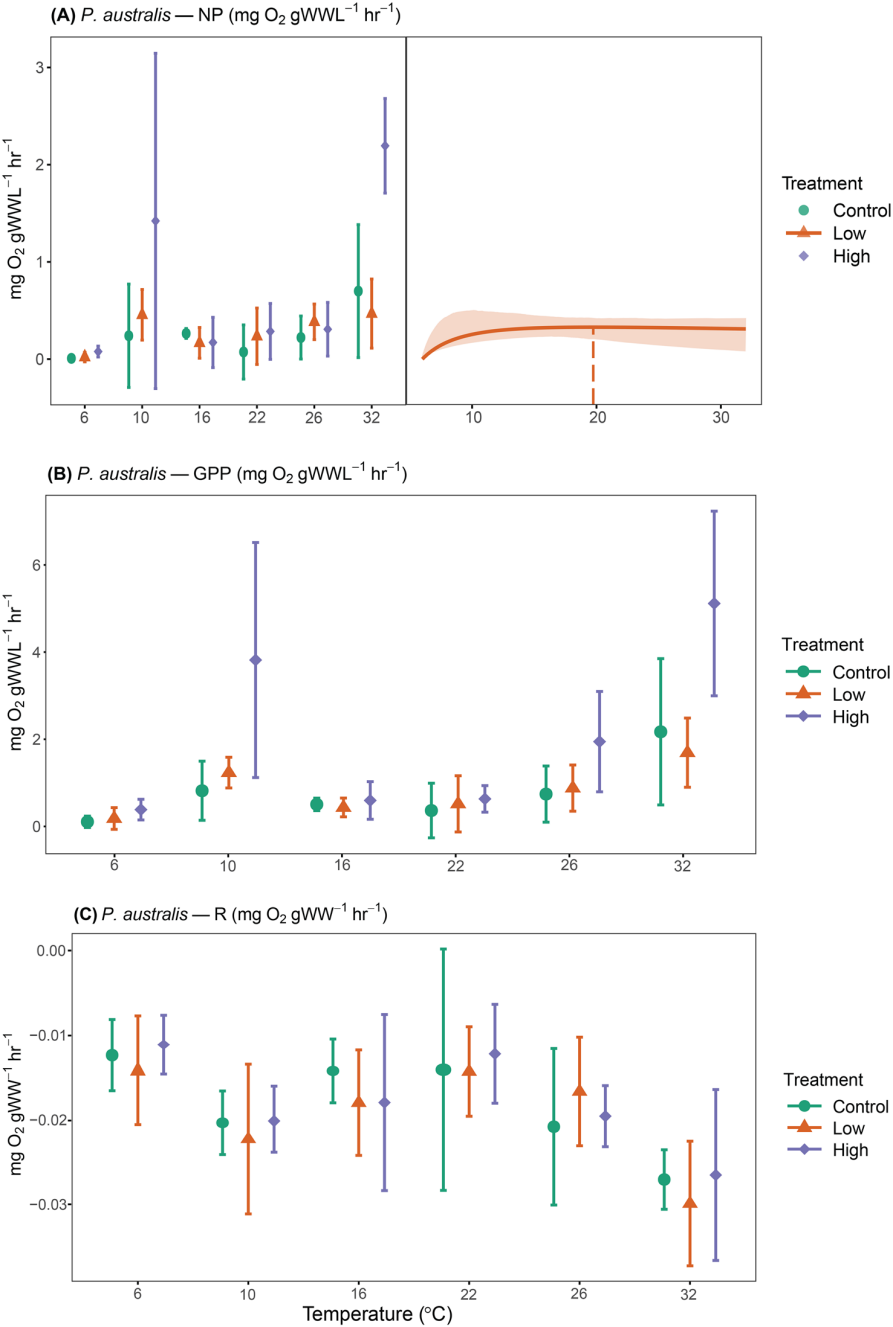
Metabolic responses of *P. australis* , showing net production (NP; A), gross primary production (GPP; B) and respiration (R; C). NP and GPP are expressed per gram wet weight of leaves (gWWL^−1^), whereas respiration is expressed per gram wet weight of plant (gWW^−1^). Left panels show treatment means ± SD at each temperature. A thermal performance curve could only be fitted for NP and is shown in the corresponding right‐hand panel. The line represents the model‐predicted mean; shaded areas indicate 95% confidence intervals, and the dashed line intersects the curve at the estimated optimal temperature (*Topt*). For GPP and R, only treatment means ± SD are shown because thermal performance curves could not be fitted.

**FIGURE 7:**
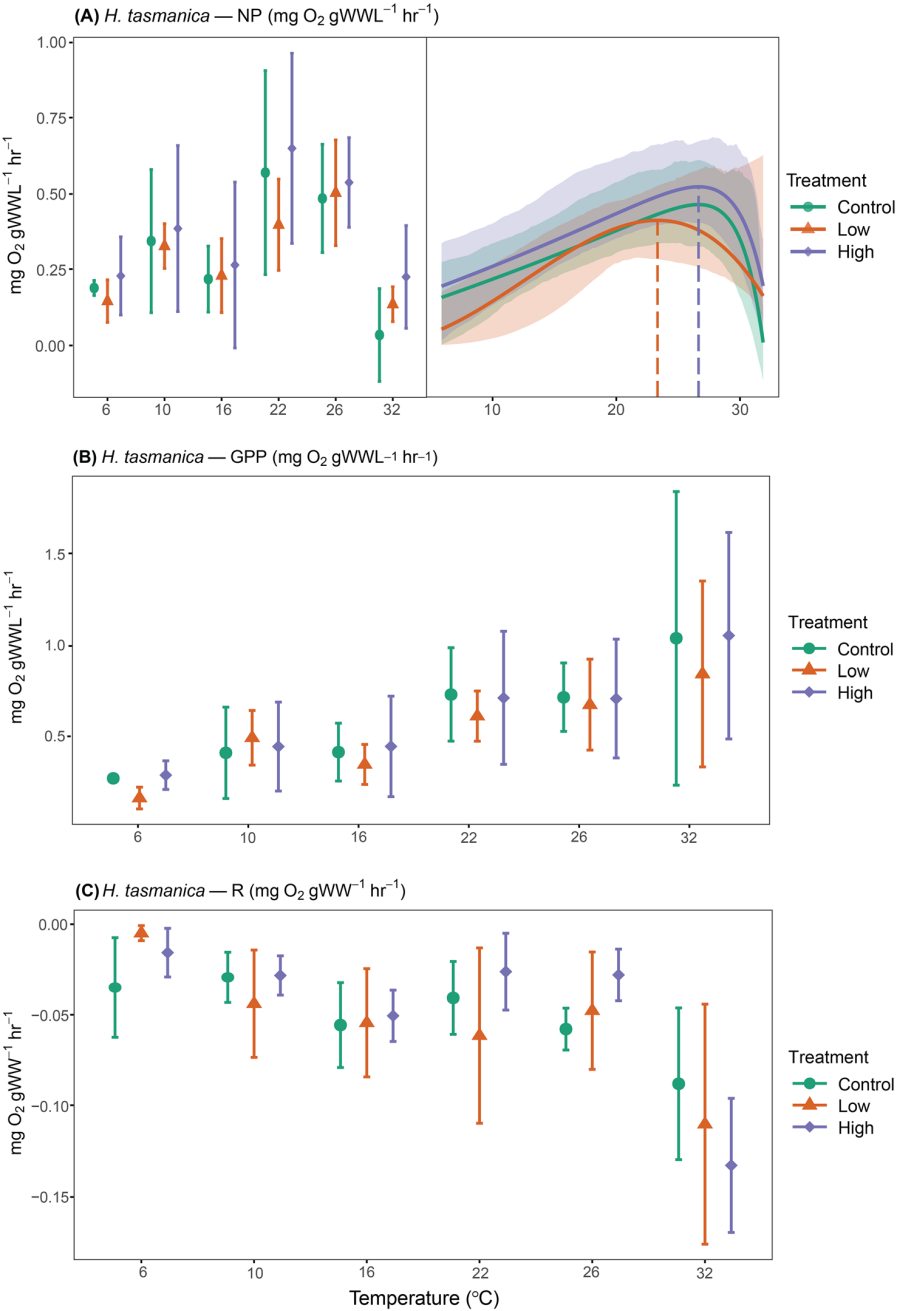
Metabolic responses of *H. tasmanica* , showing net production (NP; A), gross primary production (GPP; B) and respiration (R; C). NP and GPP are expressed per gram wet weight of leaves (gWWL^−1^), whereas respiration is expressed per gram wet weight of plant (gWW^−1^). Left panels show treatment means ± SD at each temperature. A thermal performance curve could only be fitted for NP and is shown in the corresponding right‐hand panel. The line represents the model‐predicted means, shaded areas indicate 95% confidence intervals and the dashed lines intersect the curves at the estimated optimal temperatures (*Topt*), with lines for the control and high clipping treatment overlapping. For GPP and R, only treatment means ± SD are shown because thermal performance curves could not be fitted.

The photosynthetic and respiratory responses of both seagrass species were variable across temperatures and clipping treatments. Unimodal performance curves could only be fit to the low clipping treatment of *P. australis* and the net production (NP) of *H. tasmanica* (Figures 6 and 7), while the other metabolic rates were too variable or showed a linear increase/decrease. NP and gross primary production (GPP) of *P. australis* were significantly influenced by clipping, with highly clipped plants showing a significantly higher NP than control and low‐clipped plants at 10°C and 32°C (*p* < 0.001 for both contrasts) and a higher GPP at 26°C and 32°C (*p* = 0.01 and *p* < 0.001, respectively; Table 2; Appendices 7 and 8). In *H. tasmanica* , clipping had no effect on NP and GPP (Table 2; Appendices 9 and 10). No significant differences in respiration rates between clipping treatments were found for either species.

**TABLE 2:** Estimated optimum temperatures (*Topt*) for metabolic response parameters with 95% confidence intervals (CIs) and maximum estimated rates (*Rmax*) of responses. NP and GPP are shown per gram wet weight of leaves (gWWL^−1^), R is shown per gram wet weight of plant (gWW^−1^). Asterisk * indicates estimated rates from generalised additive models [GAMs]/linear models at 32°C; no *Topt* could be determined for these parameters. Different lowercase letters indicate statistically significant differences among clipping treatment groups (CT, control treatment; LT, low clipping treatment; HT, high clipping treatment).

Species	Metabolic rate measure	Treatment	*Topt* in °C (95% CI)	*Rmax* (95% CI)
*P. australis*	NP (mg O_2_ gWWL^−1^ h^−1^)	CT	—	0.45 (0.20–0.74)*^a^
		LT	19.7 (9.5–32)	0.33 (0.25–0.50)^a^
		HT	—	1.96 (1.57–2.36)*^b^
	GPP (mg O_2_ gWWL^−1^ h^−1^)	CT	—	1.66 (0.97–2.36)*^a^
		LT	—	1.62 (0.95–2.33)*^a^
		HT	—	4.79 (3.62–5.89)*^b^
	R (mg O_2_ gWW^−1^ h^−1^)	CT	—	0.022 (0.017–0.026)*
		LT	—	0.023 (0.019–0.027)*
		HT	—	0.021 (0.017–0.025)*
*H. tasmanica*	NP (mg O_2_ gWWL^−1^ h^−1^)	CT	26.8 (24.7–30.7)	0.47 (0.32–0.60)
		LT	23.5 (17.7–26.6)	0.41 (0.28–0.57)
		HT	26.8 (22.8–30.7)	0.52 (0.40–0.63)
	GPP (mg O_2_ gWWL^−1^ h^−1^)	CT	—	1.02 (0.85–1.20)*
		LT	—	0.95 (0.77–1.12)*
		HT	—	1.04 (0.87–1.21)*
	R (mg O_2_ gWW^−1^ h^−1^)	CT	—	0.072 (0.059–0.084)*
		LT	—	0.074 (0.061–0.086)*
		HT	—	0.068 (0.056–0.081)*

### 3.3 Carbon, Nitrogen and Pigment Content

**FIGURE 8:**
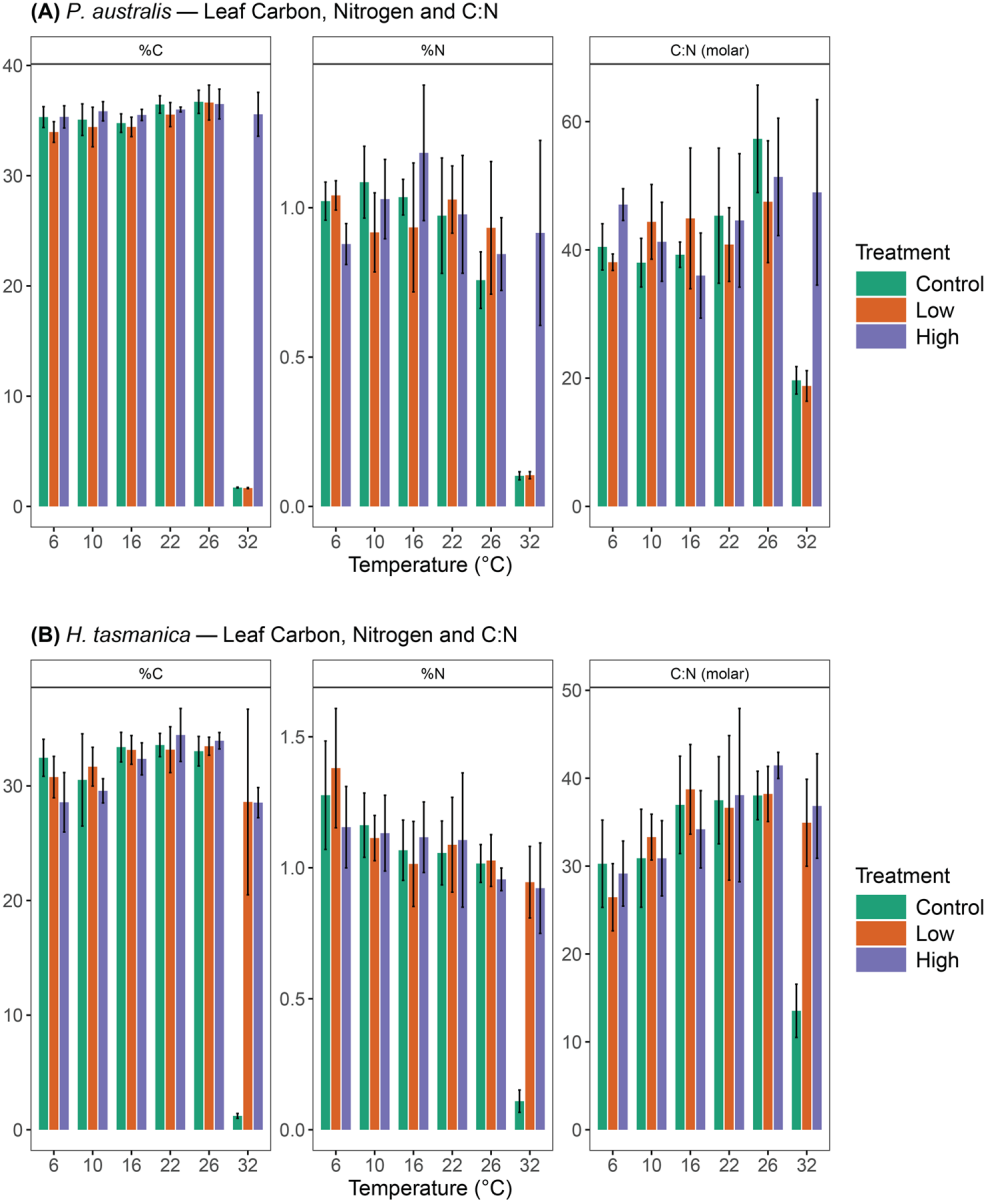
Leaf Carbon, Nitrogen (in mass percentages) and C:N (molar) of *P. australis* (A) and *H. tasmanica* (B); Bars show mean values ± SD (error bars) and are in the order of control treatment (Control: Green), low clipping treatment (Low: Orange), high clipping treatment (High: Purple).

Elemental analysis revealed a significant effect of clipping treatment on leaf carbon, nitrogen and C:N in both species (Figure 8; Appendices 11 and 12). At 32°C, highly clipped *P. australis* exhibited carbon content that was more than 20‐fold higher than in both low‐clipped and control plants (*p* < 0.01 for both contrasts). Nitrogen content at 32°C was more than 8‐fold higher in highly clipped plants compared to both low‐clipped and control plants (*p* < 0.01 for both contrasts). Similarly, C:N ratios in highly clipped plants were more than two‐fold higher than in low‐clipped plants and controls (*p* < 0.01 for both contrasts) at 32°C (Figure 8A; Appendix 13).

For *H. tasmanica* at 32°C, both highly clipped and low‐clipped plants showed significantly higher leaf nutrient concentrations compared to control plants. Carbon content was more than 23‐fold higher in both clipping treatments relative to controls (*p* < 0.01 for both contrasts). Nitrogen content at 32°C was more than 8‐fold higher in both low‐ and high‐clipped plants compared to controls (*p* < 0.01 for both contrasts), while C:N ratios were also elevated in both clipping treatments. Both highly clipped and low‐clipped *H. tasmanica* exhibited C:N ratios that were more than twofold higher than in plants of the control treatment (*p* < 0.01 for both contrasts; Figure 8B; Appendix 14).

Pigment values did not differ between clipping treatments for both species and no clear trend was visible in the total chlorophyll content or the ratio of Chl*a* to Chl*b* (Appendices 15, 16, 17).

## 4 Discussion

As climate change intensifies, the direct and indirect effects of ocean warming are impacting temperate marine macrophytes (Vergés et al. 2014, 2016; Alfonso et al. 2022; Marrone et al. 2025). Understanding how these pressures interact to influence the performance of foundation species will be critical to understanding their resilience to thermal stress. Here, we simulated herbivory (via clipping) on seagrass species to compare the thermal performance of differently sized plants across a temperature gradient that extended below and above the current range experienced by the sampled populations. We found clear evidence that clipping influences thermal performance, resulting in contrasting outcomes between species. Most notably, highly clipped *P. australis* maintained stable leaf carbon and nitrogen levels at 32°C, and despite no differences in growth across clipping treatments, highly clipped *P. australis* demonstrated significantly higher rates of net production (NP) and gross primary production (GPP) at 32°C. Clipped *H. tasmanica* also showed higher contents of leaf C and N at 32°C; however, there was no evidence for higher photosynthetic performance relative to control plants, and clipped plants displayed a lower *Topt* for growth rates compared to control plants. Our findings reveal variation among species and across performance metrics within species in their responses to simulated herbivory, providing novel insights into the resilience of seagrass to ongoing ocean warming and extreme events.

We hypothesised that simulated herbivory would decrease individual cost of tissue maintenance, allowing for increased resource investment in the protection of the remaining tissue and thereby improving thermal performance. The combination of significantly higher metabolic rates and leaf C and N in highly clipped *P. australis* at 32°C, relative to low‐clipped and control plants, provides support for this hypothesis, although this response did not translate into increased growth. Plants of the genus *Posidonia* are known for the great storage capacities of their rhizomes (Alcoverro et al. 2001; O'Brien et al. 2018). Our results suggest that clipping enabled sustained nutrient resource allocation to the leaves, throughout the exposure to thermal stress. In contrast, larger leaf size may have exhausted nutrient supplies. Pigment concentrations (Chl*a*, Chl*b*) and ratios (Chl*a*/Chl*b*) did not differ between clipping treatments, indicating that resources were not invested into adapting the photosynthetic apparatus. Instead, additional resources may have been allocated to the production of heat‐protection enzymes (Reynolds et al. 2016; Marín‐Guirao et al. 2017; Traboni et al. 2018), soluble carbohydrates (Gu et al. 2012; Marín‐Guirao et al. 2018; Moreno‐Marín et al. 2018), or herbivory‐related defence and repair pathways (Vergés et al. 2008; Egea and Jiménez‐Ramos 2025), resulting in higher contents of leaf C and N.

Although the metabolic rates and leaf nutrient content of highly clipped *P. australis* at 32°C differed to low‐clipped and control plants, growth—as an aggregate measure of thermal performance for the entire experimental duration—did not differ between clipping treatments, and all treatment groups showed low growth rates at 32°C. Terrestrial plants are known to allocate resources to thermal protection and damage repair under heat stress, often at the expense of other metabolic processes such as growth (Wahid et al. 2007). A similar pattern has been reported for the temperate seagrass *P. oceanica* , which increased carbohydrate storage at the expense of leaf growth under elevated temperatures (Marín‐Guirao et al. 2018), while short‐term heat exposure has been shown to activate molecular and physiological protection mechanisms that may not immediately translate into growth (Marín‐Guirao et al. 2017; Ruocco et al. 2019). Our results therefore suggest that highly clipped *P. australis* maintained stronger physiological and biochemical functioning at 32°C, potentially because fewer resources were required to maintain the remaining leaf biomass, leaving a greater proportion available for tissue protection and repair. This apparent physiological advantage did not translate into higher growth during the 28‐day experiment and may require a longer exposure or recovery period to become evident. Nevertheless, our observations align with those of Bennett, Alcoverro, et al. (2022), who found that cool‐edge *P. oceanica* exposed to both extreme thermal anomalies and severe grazing maintained high survival and subsequently recovered during a year‐long field experiment.

In contrast to *P. australis* , *H. tasmanica* showed signs of decreased thermal resilience with clipping. Although clipped *H. tasmanica* similarly retained higher leaf C and N at 32°C while control plants showed a sharp decline, photosynthetic rates and their *Topt* did not differ between treatments. Furthermore, clipping reduced *Topt* for growth, resulting in lower growth of clipped plants at higher temperatures. These patterns suggest higher costs of leaf repair at elevated temperatures (Marín‐Guirao et al. 2018), without the capacity to translate the increased nutrient contents (relative to leaf size) into increased photosynthetic rates.

These observed species‐specific differences in the effect of simulated herbivory on thermal resilience may appear counterintuitive given the differences in each species overall body size: reducing leaf size did not enhance the thermal resilience in the smaller‐bodied *H. tasmanica* , yet it did in the larger‐bodied *P. australis* . Considering the different evolutionary histories and biogeography of the two species helps to put these observations into context. *P. australis* and *H. tasmanica* belong to distinct temperate seagrass families with differing latitudinal distributions and diversification history (Tuya et al. 2026). *Posidonia* is an ancient lineage with extraordinarily low evolutionary rates (Aires et al. 2011) and Tethyan origins under climates ~2°C–3°C warmer than present (Bianchi et al. 2012). Under these conditions, *Posidonia* co‐occurred with marine megaherbivores (sirenians, sea turtles) known to feed predominately on seagrasses (Vélez‐Juarbe 2014; Esteban et al. 2020; Chiarenza et al. 2023). This is reflected in the current biogeographical range of *P. australis* , occurring from cold‐temperate to subtropical waters (Bass Strait to Shark Bay; Commonwealth of Australia 2006) with high abundances of megaherbivores and high rates of herbivory (Preen et al. 1997; Heithaus et al. 2005; Burkholder et al. 2013). In contrast, *Heterozostera* diversified more recently within the Southern Ocean's cold‐temperate climate (Sullivan and Short 2023) exhibiting shorter generation times (Kato et al. 2003), linked to faster evolutionary rates (Smith and Donoghue 2008). Historical evidence for the occurrence of megaherbivores across the biogeographical range of *H. tasmanica* is sparse (Pledge 2006; Chiarenza et al. 2023), and no resident megaherbivore populations are documented within this range (Marsh and Sobtzick 2019; Hays et al. 2025). Moreover, herbivory rates are generally lower in cold‐temperate marine environments (Floeter et al. 2005; Vergés et al. 2018). Together, these differences suggest that *P. australis* may have evolved mechanisms to better cope with increased thermal stress and herbivory pressure, whereas *H. tasmanica*, likely exposed to comparatively lower levels of these pressures, appears more sensitive despite its smaller size.

One potential mechanism which may facilitate *P. australis* to better cope with increased temperature is the storage capacity of its rhizomes. *P. australis* and its congeneric species like *P. oceanica* , are known to maintain substantial below‐ground carbon stores and to translocate resources along rhizomes (Alcoverro et al. 2001; O'Brien et al. 2018). *H. tasmanica* has a much smaller rhizome suggesting limited internal storage of non‐structural carbohydrates and nutrients compared to *P. australis*, and providing a plausible explanation for the higher survival of *P. australis* at 32°C. The sharp decline in survival of *H. tasmanica* at 32°C across all clipping treatments indicates that this temperature exceeded the capacity of this species to maintain essential physiological functions. This aligns with reports of higher survival in *P. australis* relative to *Amphibolis antarctica*, which has a similarly smaller below‐ground storage capacity to *H. tasmanica* (Cambridge et al. 2012), during an intense marine heatwave (Fraser et al. 2014; Strydom et al. 2020). These inter‐specific differences have clear implications for future warming scenarios: during marine heatwave events, species with smaller rhizomes such as *H. tasmanica* or *A. antarctica* may face higher risks than *Posidonia* species.

The relative impacts of future warming on different species may also vary between populations across each species' range. Intra‐specific variation in thermal performance can arise from acclimatisation history, genetic differentiation and phenotypic plasticity (Marín‐Guirao et al. 2016; Alsuwaiyan et al. 2021; Pazzaglia et al. 2021), as frequently shown by studies comparing warm‐edge to cool‐edge populations (McCoy and Widdicombe 2019; Bennett, Alcoverro, et al. 2022; Bennett, Vaquer‐Sunyer, et al. 2022; Shi et al. 2026; Butler et al. 2026). In this study, both, *P. australis* and *H. tasmanica* were examined at the cool edge of their ranges (Kuo 2005; Garthwin et al. 2024). Our estimated *Topt* values may therefore be lower than those of warm‐edge conspecifics, as shown by Bulthuis and Woelkerling (1983); reviewed in Lee et al. (2007), who reported a photosynthetic *Topt* of 30°C for *H. tasmanica* in Victoria. Similarly, *P. australis* in Shark Bay (warm edge) has persisted through ~30°C conditions for over 10 weeks (Strydom et al. 2020). Because our experiment did not impose a prolonged heatwave, nor did we track post‐stress recovery, we cannot directly compare our data with these warm‐edge populations. Nevertheless, the high survival of *P. australis* over 4 weeks at 32°C indicates substantial heat tolerance despite typical temperature maxima near 21°C at the local site (Figure 2B). To gain a more complete understanding of the thermal resilience and the impact of herbivory on a species, a priority for future research should be the comparison of different populations within a species (i.e., warm‐edge vs. cool‐edge) and the use of field‐based studies or in‐situ transplants in areas with high herbivory pressure.

Experimental studies aim to apply treatments that reflect the ecological conditions experienced by species in their natural habitats. However, relative to field estimates, *P. australis* in our experiment exhibited lower growth rates and higher C:N values (Walker and McComb 1988; Garthwin et al. 2024). For *H. tasmanica* , leaf extension was broadly comparable, but leaf %N was lower than in previous field studies (Bulthuis and Woelkerling 1983; Campbell and Miller 2002). Together, these comparisons indicate that the absolute rates/values obtained under laboratory conditions may be lower than those of previous field observations, likely reflecting experimental artefacts (e.g., nutrient availability, artificial light/flow, containment effects, absence of sediment in experimental aquaria) or differences between local environmental conditions at the collection sites compared to previous field studies. A further consideration is that, while clipping provides a standardised and widely used approach to simulate biomass loss from herbivory (Sanmartí et al. 2014; Rodriguez et al. 2022), it does not fully reproduce the ecological complexity of natural grazing (e.g., selective grazing, repeated grazing; Lehtilä and Boalt 2008; Jiménez‐Ramos et al. 2024). Accordingly, our results are best interpreted as relative differences among treatments rather than absolute values expected in wild populations exposed to naturally occurring herbivory.

To our knowledge, this is the first study to experimentally quantify the effects of simulated herbivory on seagrass thermal performance across a broad temperature gradient. To better understand the response of marine macrophytes to herbivory and increased water temperatures we suggest three priorities for future work: (i) pair measures of thermal performance with further biochemical measures of heat protection; (ii) quantify and, where appropriate, manipulate dissolved nutrient concentrations, especially nitrogen, to reflect local conditions and generate ecologically relevant responses; and (iii) investigate the reverse pathway by testing how warming events, including marine heatwaves, may alter macrophyte resistance or tolerance to subsequent herbivory.

Where previous studies frame herbivory as a stressor that exacerbates warming impacts on macrophytes (Vergés et al. 2016; Martínez‐Crego et al. 2021), our results recast the role of herbivory and suggest that in some contexts, herbivory may increase thermal resilience of seagrasses by shifting resource allocation, as demonstrated for *P. australis* . In contrast, *H. tasmanica* appears more sensitive, with clipping reducing thermal performance by lowering the optimal temperature for growth. Under future climates, *P. australis* could be a relative winner, whereas *H. tasmanica* may face compounding risks.

## Author Contributions

**Niclas Einert:** conceptualization (supporting), data curation (lead), formal analysis (lead), investigation (lead), methodology (supporting), project administration (lead), software (lead), validation (equal), visualization (lead), writing – original draft (lead), writing – review and editing (equal). **Claire Butler:** conceptualization (supporting), formal analysis (supporting), investigation (supporting), methodology (supporting), resources (equal), software (supporting), supervision (equal), validation (equal), writing – review and editing (equal). **Amanda K. Pettersen:** conceptualization (supporting), funding acquisition (equal), investigation (supporting), methodology (supporting), project administration (supporting), resources (equal), supervision (equal), validation (equal), writing – review and editing (equal). **Scott Bennett:** conceptualization (lead), funding acquisition (equal), methodology (lead), resources (equal), supervision (equal), validation (equal), writing – review and editing (equal).

## Funding

This work was supported by the German Academic Exchange Service, Stipendien fuer ein Masterstudium im Ausland. Australian Research Council, DE200100900. Institute for Marine and Antarctic Studies, Honours/Masters Research Project Grant.

## Conflicts of Interest

The authors declare no conflicts of interest.

## Data Availability

The study protocol was registered on the Open Science Framework (OSF). All data and code from this study are available in the OSF project maintained by NE under https://doi.org/10.17605/OSF.IO/T4SNX.

## Appendix 1

Heating/chilling equipment used to keep the water baths at their respective treatment temperatures.

**Table:**

Tank (treatment temperature)	Heating/Chilling unit
T1 (6°C)	Kegland G20 Glycol chiller (Kegland Home Brew Community Group, Australia)
T2 (10°C)	Teco TK500 aquarium chiller (TECO Refrigeration Technologies, Italy)
T3 (16°C)	—
T4 (22°C)	AquaOne TH 100 aquarium heater (Kong's Pty Limited, Australia)
T5 (26°C)	IKS aquastar temperature control unit (IKS Computersysteme GMBH, Germany) 2 × Schego Titanium aquarium heater 300 W (SCHEGO Schemel & Goetz GmbH & Co KG, Germany)
T6 (32°C)	IKS aquastar temperature control unit (IKS Computersysteme GMBH, Germany) 2 × Schego Titanium aquarium heater 600 W (SCHEGO Schemel & Goetz GmbH & Co KG, Germany)

## Appendix 2

Temperature (°C) of water baths over experimental duration showing ramping and treatment period; Dotted lines indicate start and finish of experiment, dates are: T1: 30.04.—02.06.2025, T2: 30.04.—31.05.2025, T3: 30.04.—28‐05.2025, T4: 30.04.—31.05.2025, T5: 30.04.—02.06.2025, T6: 30.04.—04.06.2025; No data for T5 (26°C) due to battery issues with temperature logger.

## Appendix 3

Nutrient values (μg L^−1^) from the local area of the experimental facility, sampled on 09.09.2025, at the same outlet that was used for the water in the experimental aquaria (bags). Number of samples: *n* = 4.

**Table:**

Analyte	Mean value (μg/L)
Ammonium	27.6
Nitrate + Nitrite	329.75
Phosphate	18.45

## Appendix 4

Akaike Information Criterion (AIC), Bayesian Information Criterion (BIC) and delta AIC for each of the thermal performance curve (TPC) models fitted to the three growth responses of *P. australis* . Only models with a deltaAIC < 2 are shown. Models highlighted in bold indicate those selected as best fit model.

**Table:**

** *P. australis* —Growth (mg d** ^**−1**^ **)—Control treatment**			
**Model**	**AIC**	**BIC**	**deltaAIC**
Modified_Gaussian	157.5638	164.5698	0
**Gaussian**	**158.1257**	**163.7305**	**0.5618651**
Weibull	158.2317	165.2377	0.6678263
lrf	159.272	166.278	1.7081617
** *P. australis* —Growth (mg d** ^**−1**^ **)—Low clipping treatment**			
**Model**	**AIC**	**BIC**	**deltaAIC**
**Gaussian**	**181.7378**	**187.3426**	**0**
lrf	181.9512	188.9572	0.2133712
yanhunt	182.8256	188.4304	1.0878143
Weibull	183.2655	190.2715	1.5276889
Modified_Gaussian	183.6541	190.6601	1.9162467
** *P. australis* —Growth (mg d** ^**−1**^ **)—High clipping treatment**			
**Model**	**AIC**	**BIC**	**deltaAIC**
lrf	162.7783	169.7843	0
**Weibull**	**163.7008**	**170.7068**	**0.9224414**
Gaussian	164.0913	169.6961	1.3129653
Modified_Gaussian	164.6721	171.6781	1.8937838
** *P. australis* —Growth (RGR d** ^**−1**^ **)—Control treatment**			
**Model**	**AIC**	**BIC**	**deltaAIC**
Weibull	3.968859	10.97485	0
**Gaussian**	**4.507773**	**10.11256**	**0.53891368**
Modified_Gaussian	4.68251	11.6885	0.71365033
lrf	5.606031	12.61202	1.63717144
** *P. australis* —Growth (RGR d** ^**−1**^ **)—Low clipping treatment**			
**Model**	**AIC**	**BIC**	**deltaAIC**
lrf	33.525632	40.53162	0
**Gaussian**	**34.072617**	**39.67741**	**0.54698495**
yanhunt	34.656429	40.26122	1.13079757
Weibull	35.384939	42.39093	1.85930699
** *P. australis* —Growth (RGR d** ^**−1**^ **)—High clipping treatment**			
**Model**	**AIC**	**BIC**	**deltaAIC**
**Gaussian**	**13.387558**	**18.99235**	**0**
Modified_Gaussian	13.933435	20.93942	0.54587729
Weibull	14.806974	21.81296	1.41941695
** *P. australis* —Growth (mm d** ^**−1**^ **)—Control treatment**			
**Model**	**AIC**	**BIC**	**deltaAIC**
lrf	135.4832	142.4892	0
Modified_Gaussian	135.7126	142.7185	0.2293933
**Weibull**	**135.8639**	**142.8699**	**0.3807216**
** *P. australis* —Growth (mm d** ^**−1**^ **)—Low clipping treatment**			
**Model**	**AIC**	**BIC**	**deltaAIC**
**Gaussian**	**134.0742**	**139.679**	**0**
Modified_Gaussian	135.35	142.356	1.2758154
Weibull	135.6597	142.6657	1.5854761
** *P. australis* —Growth (mm d** ^**−1**^ **)—High clipping treatment**			
**Model**	**AIC**	**BIC**	**deltaAIC**
Modified_Gaussian	114.3259	121.3319	0
**Gaussian**	**114.5398**	**120.1446**	**0.2139295**
Weibull	115.5464	122.5524	1.2205397

## Appendix 5

Akaike Information Criterion (AIC), Bayesian Information Criterion (BIC) and delta AIC for each of the Thermal Performance Curve (TPC) models fitted to the three growth responses of *H. tasmanica* . Only models with ΔAIC < 2 are shown, except where the selected model exceeded this threshold (refer to model selection criteria in chapter 2.7). Models highlighted in bold indicate those selected as best fit model.

**Table:**

** *H. tasmanica* —Growth (mg d** ^**−1**^ **)—Control treatment**			
**Model**	**Model**	**Model**	**Model**
**yanhunt**	**146.715**	**152.3198**	**0**
quadratic	147.5552	153.16	0.8402662
Weibull	148.6215	155.6275	1.9065021
lrf	148.6379	155.6439	1.9229561
** *H. tasmanica* —Growth (mg d** ^**−1**^ **)—Low clipping treatment**			
**Model**	**AIC**	**BIC**	**deltaAIC**
**yanhunt**	**153.2324**	**158.8372**	**0**
quadratic	153.4802	159.085	0.2477829
Thomas	154.9883	161.9943	1.7559072
Modified_Gaussian	155.0166	162.0225	1.7841563
lrf	155.1023	162.1083	1.8698697
** *H. tasmanica* —Growth (mg d** ^**−1**^ **)—High clipping treatment**			
**Model**	**AIC**	**BIC**	**deltaAIC**
Modified_Gaussian	169.6161	176.6221	0
Weibull	172.2764	179.2824	2.6603449
**yanhunt**	**172.3646**	**177.9694**	**2.7485616**
** *H. tasmanica* —Growth (RGR d** ^**−1**^ **)—Control treatment**			
**Model**	**AIC**	**BIC**	**deltaAIC**
**yanhunt**	**95.307709**	**100.9125**	**0**
quadratic	96.049963	101.65475	0.74225415
Gaussian	96.509938	102.11473	1.20222928
lrf	96.929054	103.93504	1.6213448
** *H. tasmanica* —Growth (RGR d** ^**−1**^ **)—Low clipping treatment**			
**Model**	**AIC**	**BIC**	**deltaAIC**
**quadratic**	**101.413092**	**107.01788**	**0**
yanhunt	101.444542	107.04933	0.03145069
lrf	102.137994	109.14398	0.72490226
** *H. tasmanica* —Growth (RGR d** ^**−1**^ **)—High clipping treatment**			
**Model**	**AIC**	**BIC**	**deltaAIC**
Weibull	112.320983	119.32697	0
Modified_Gaussian	113.040321	120.04631	0.71933832
**yanhunt**	**113.460747**	**119.06554**	**1.13976463**
quadratic	113.651668	119.25646	1.33068532
** *H. tasmanica* —Growth (mm d** ^**−1**^ **)—Control treatment**			
**Model**	**AIC**	**BIC**	**deltaAIC**
**yanhunt**	**136.7103**	**142.315**	**0**
Weibull	137.7024	144.7083	0.9920975
** *H. tasmanica* —Growth (mm d** ^**−1**^ **)—Low clipping treatment**			
**Model**	**AIC**	**BIC**	**deltaAIC**
Modified_Gaussian	154.6762	161.6822	0
lrf	159.1391	166.1451	4.4629444
Weibull	159.9992	167.0052	5.3230529
**yanhunt**	**160.3437**	**165.9485**	**5.6675176**
** *H. tasmanica* —Growth (mm d** ^**−1**^ **)—High clipping treatment**			
**Model**	**AIC**	**BIC**	**deltaAIC**
**yanhunt**	**145.7893**	**151.3941**	**0**
quadratic	146.3044	151.9092	0.5151195
Modified_Gaussian	146.6462	153.6522	0.8568962
lrf	147.7374	154.7434	1.9480415

## Appendix 6

Pairwise contrasts of bootstrapped estimated optimum temperatures (Topt) and maximum rates (Rmax) between clipping treatments, for the three growth responses tested, with 95% confidence intervals (CI); CT, control treatment; LT, low clipping treatment; HT, high clipping treatment; significant differences (*p* < 0.05) are highlighted in bold.

**Table:**

Species	Growth measure	Parameter	Contrast	Estimate	Lower CI	Upper CI	*p*
*P. australis*	mg d^−1^	*Topt*	CT–LT	−1.739	−4.478	1.663	0.920
			CT–HT	−0.623	−3.278	3.795	0.924
			LT–HT	1.135	−2.055	5.433	0.924
		*Rmax*	CT–LT	−2.718	−9.959	2.859	0.813
			CT–HT	0.727	−5.551	4.571	0.813
			LT–HT	3.406	−3.377	10.462	0.813
	RGR d^−1^	*Topt*	CT–LT	−1.481	−4.346	2.560	0.801
			CT–HT	0.619	−1.993	3.717	0.801
			LT–HT	2.109	−1.764	5.218	0.685
		*Rmax*	CT–LT	0.000	−0.001	0.000	1.000
			CT–HT	0.000	−0.001	0.000	1.000
			LT–HT	0.000	−0.001	0.001	1.000
	mm d^−1^	*Topt*	CT–LT	−3.102	−5.776	0.280	0.216
			CT–HT	−0.761	−3.197	3.295	0.674
			LT–HT	2.352	−0.388	6.132	0.216
		*Rmax*	CT–LT	0.006	−3.239	2.702	0.997
			CT–HT	1.522	−0.761	3.630	0.593
			LT–HT	1.516	−1.304	4.787	0.628
*H. tasmanica*	mg d^−1^	*Topt*	CT–LT	0.618	−1.354	2.702	0.565
			**CT**–**HT**	**4.551**	**1.643**	**8.091**	**< 0.001**
			**LT**–**HT**	**3.915**	**1.148**	**7.301**	**0.003**
		*Rmax*	CT–LT	−1.777	−3.905	0.437	0.356
			CT–HT	−1.782	−4.148	0.379	0.356
			LT–HT	−0.034	−2.823	2.606	0.983
	RGR d^−1^	*Topt*	**CT**–**LT**	**1.812**	**0.186**	**3.651**	**0.024**
			**CT**–**HT**	**4.242**	**1.695**	**7.112**	**< 0.001**
			**LT**–**HT**	**2.369**	**0.365**	**4.787**	**0.019**
		*Rmax*	CT–LT	−0.001	−0.002	0.000	0.119
			**CT**–**HT**	**−0.001**	**−0.002**	**0.000**	**0.019**
			LT–HT	0.000	−0.001	0.001	0.607
	mm d^−1^	*Topt*	CT–LT	1.920	0.004	3.910	0.099
			**CT**–**HT**	**3.039**	**1.325**	**4.947**	**< 0.001**
			LT–HT	1.118	−1.066	3.348	0.329
		*Rmax*	CT–LT	1.876	−0.777	4.515	0.219
			**CT**–**HT**	**4.007**	**1.933**	**6.130**	**< 0.001**
			LT–HT	2.127	−0.426	4.718	0.219

## Appendix 7

Generalised Additive Model (GAM) and Linear Mixed Model (LMM) output tables testing for the effect of clipping treatment on the net production (NP), gross primary production (GPP) and respiration rates (*R*) of *P. australis* . GAMs were used for NP and GPP with parametric coefficients giving linear effects (intercept and contrasts between clipping treatments) at the treatment temperatures; smooth terms model non‐linear temperature responses for each clipping treatment (CT, control treatment; LT, low clipping treatment; HT, high clipping treatment) with effective degrees of freedom (*edf*) indicating curve complexity; significance of terms given for parametric coefficients (*t/z, Pr(>|t/z|)*) and smooth terms (reference degrees of freedom [*Ref.df*], *F/Chi.sq*, *p*‐value). An LMM was used for R, showing the ANOVA output of degrees of freedom (*Df*), F‐statistic for the fixed effect (*F*) and *p*‐value (*Pr(>F)*).

**Table:**

*P. australis* —NP (mg O_2_ gWWL^−1^ h^−1^)				
Parametric coefficients	Estimate	Std. Error	*t*	Pr(>|t|)
(Intercept)	0.39288	0.04737	8.294	2.07E‐12
Treatment1	−0.15859	0.06699	−2.367	0.0203
Treatment2	−0.12502	0.06699	−1.866	0.0656
**Smooth terms**	**edf**	**Ref.df**	***F***	***p***
s(TempTreat_num):CT	1.066	1.129	2.37	0.11
s(TempTreat_num):LT	1	1	1.11	0.295
s(TempTreat_num):HT	4.518	4.893	13.73	< 2e‐16

*P. australis* —GPP (mg O_2_ gWWL^−1^ h^−1^)				
Parametric coefficients	Estimate	Std. Error	z	Pr(>|z|)
(Intercept)	0.97205	0.06821	14.252	< 2e‐16
Treatment1	−0.4112	0.0843	−4.878	1.07E‐06
Treatment2	−0.39991	0.08308	−4.814	1.48E‐06
(Intercept)0.1	−0.654	0.07776	−8.411	< 2e‐16
**Smooth terms**	**edf**	**Ref.df**	**Chi.sq**	***p***
s(TempTreat_num)	1.1145571	1.217473	7.996	0.00636
s(TempTreat_num): CT	1.0007679	1.001502	0.081	0.7775
s(TempTreat_num): LT	0.0008954	0.001749	0	0.98983
s(TempTreat_num): HT	4.5595517	4.900585	49.783	< 2e‐16
s.1(TempTreat_num)	4.7839197	4.97638	113.215	< 2e‐16

*P. australis* —R (mg O_2_ gWW^−1^ h^−1^)			
	Df	*F*	Pr(>F)
(Intercept)	1	26.3708	1.73E‐06
TempTreat_num	1	16.5244	0.0001059
Treatment	2	0.2287	0.7960487
Residuals	86		

## Appendix 8

Tukey's Honestly Significant Difference (HSD) posthoc test results showing pairwise contrasts of the effect of clipping treatment on the net production (NP) and gross primary production (GPP) rates of *P. australis* across temperatures; CT, control treatment; LT, low clipping treatment; HT, high clipping treatment. Standard errors (SE) quantify uncertainty around the estimated differences between contrasts, *t.ratio* and *z* show *Estimate* divided by SE with respective degrees of freedom (df) for *t.ratio*. Significant differences between clipping treatments (*p* < 0.05) are highlighted in bold.

**Table:**

*P. australis* —NP (mg O_2_ gWWL^−1^ h^−1^)						
Temperature	Contrast	Estimate	SE	df	*t*. ratio	*p*
6°C	CT–LT	−0.102602	0.202	80.4	−0.508	1
	CT–HT	−0.094334	0.245	80.4	−0.385	1
	LT–HT	0.008268	0.244	80.4	0.034	1
10°C	CT–LT	−0.082831	0.161	80.4	−0.513	0.61
	CT–HT	−1.064683	0.222	80.4	−4.793	**< 0.001**
	LT–HT	−0.981853	0.222	80.4	−4.424	**< 0.001**
16°C	CT–LT	−0.053085	0.123	80.4	−0.43	1
	CT–HT	−0.062244	0.211	80.4	−0.295	1
	LT–HT	−0.009159	0.21	80.4	−0.044	1
22°C	CT–LT	−0.019984	0.126	80.4	−0.159	1
	CT–HT	0.083409	0.207	80.4	0.402	1
	LT–HT	0.103394	0.206	80.4	0.502	1
26°C	CT–LT	0.00628	0.15	80.4	0.042	1
	CT–HT	−0.000472	0.217	80.4	−0.002	1
	LT–HT	−0.006752	0.217	80.4	−0.031	1
32°C	CT–LT	0.050841	0.209	80.4	0.243	0.81
	CT–HT	−1.514865	0.249	80.4	−6.074	**< 0.001**
	LT–HT	−1.565706	0.248	80.4	−6.318	**< 0.001**

*P. australis* —GPP (mg O_2_ gWWL^−1^ h^−1^)					
Temperature	Contrast	Estimate	SE	*z*	*p*
6°C	LT‐CT	0.0618	0.108	0.573	0.57
	HT‐CT	0.248	0.109	2.27	0.07
	HT‐LT	0.186	0.109	1.71	0.18
10°C	LT‐CT	0.0214	0.127	0.168	0.87
	HT‐CT	1.16	0.479	2.42	0.05
	HT‐LT	1.14	0.477	2.38	0.05
16°C	LT‐CT	−0.00608	0.132	−0.0462	0.98
	HT‐CT	0.137	0.148	0.927	0.98
	HT‐LT	0.143	0.147	0.978	0.98
22°C	LT‐CT	0.0232	0.167	0.139	1.00
	HT‐CT	0.137	0.219	0.624	1.00
	HT‐LT	0.114	0.218	0.522	1.00
26°C	LT‐CT	0.0221	0.244	0.0903	0.93
	HT‐CT	0.979	0.313	3.12	**0.01**
	HT‐LT	0.956	0.312	3.07	**0.01**
32°C	LT‐CT	−0.0247	0.478	−0.0516	0.96
	HT‐CT	2.85	0.679	4.2	**< 0.001**
	HT‐LT	2.88	0.675	4.26	**< 0.001**

## Appendix 9

Pairwise contrasts between clipping treatments of *H. tasmanica* comparing estimated optimum temperatures (*Topt*) and maximum rates (*Rmax*) of net production (NP) with 95% confidence intervals (CIs); CT, control treatment; LT, low clipping treatment; HT, high clipping treatment.

**Table:**

*H. tasmanica* —NP (mg O_2_ gWWL^−1^ h^−1^)					
Parameter	Contrast	Estimate	Lower CI	Upper CI	*p*
*Topt*	LT–CT	−1.847	−7.034025	2.678	1
	HT–CT	0.245	−5.055	5.162	1
	HT–LT	2.065	−3.262	7.577	1
*Rmax*	LT–CT	−0.059	−0.232	0.115	0.820
	HT–CT	0.070	−0.091	0.242	0.820
	HT–LT	0.131	−0.032	0.301	0.361

## Appendix 10

Generalised Additive Model (GAM) and Linear Mixed Model (LMM) output tables testing for the effect of clipping treatment on the gross primary production (GPP) and respiration (R) rates of *H. tasmanica* . Refer to Appendix 8 for detail on the statistical tests; a linear GAM was used to test for the effect of clipping treatments on GPP rates, hence no smooth term statistics are shown.

**Table:**

*H. tasmanica* —GPP (mg O_2_ gWWL^−1^ h^−1^)				
Parametric coefficients	Estimate	Std. Error	*t*	Pr(>|t|)
(Intercept)	0.614477	0.037484	16.393	< 2e‐16
Treatment1	0.018359	0.053222	0.345	0.731
Treatment2	−0.057454	0.053222	−1.08	0.285
Temp_c	0.029304	0.004534	6.463	2.04E‐08

*H. tasmanica* —R (mg O_2_ gWW^−1^ h^−1^)			
	Df	*F*	Pr(>F)
(Intercept)	1	1.8926	0.1725
TempTreat_num	1	30.308	3.76E‐07
Treatment	2	0.2458	0.7826
Residuals	86		

## Appendix 11

Generalised Additive Model (GAM) output tables analysing the effect of clipping treatment on the leaf nutrient contents of *P. australis*. Refer to Appendix 8 for detail on the statistical tests.

**Table:**

** *P. australis* —Leaf Carbon (mass %)**				
**Parametric coefficients**	**Estimate**	**Std. Error**	***t***	**Pr(>|t|)**
(Intercept)	−1.11393	0.008003	−139.182	< 2e‐16
TreatmentLow	−0.026166	0.011319	−2.312	0.0208
TreatmentHigh	0.538806	0.011319	47.604	< 2e‐16
**Smooth terms**	**edf**	**Ref.df**	***F***	***p***
s(Temp_num): TreatmentControl	4.9932216	5	22105.273	< 2e‐16
s(Temp_num): TreatmentLow	4.9936463	5	22026.086	< 2e‐16
s(Temp_num): TreatmentHigh	0.503916	5	1.008	0.158
** *P. australis* —Leaf Nitrogen (mass %)**				
**Parametric coefficients**	**Estimate**	**Std. Error**	***z***	**Pr(>|z|)**
(Intercept)	0.829422	0.027473	30.19	< 2e‐16
TreatmentLow	−0.003207	0.038853	−0.083	0.934432
TreatmentHigh	0.142158	0.038853	3.659	0.000464
**Smooth terms**	**edf**	**Ref.df**	**Chi.sq**	***p***
s(Temp_num): TreatmentControl	3.432	4.074	37.189	< 2e‐16
s(Temp_num): TreatmentLow	4.068	4.631	29.74	< 2e‐16
s(Temp_num): TreatmentHigh	3.276	3.915	3.601	0.013
** *P. australis* —Leaf C:N (molar)**				
**Parametric coefficients**	**Estimate**	**Std. Error**	***t***	**Pr(>|t|)**
(Intercept)	3.48077	0.03038	114.564	< 2e‐16
TreatmentLow	−0.02129	0.04297	−0.495	0.62176
TreatmentHigh	0.14673	0.04297	3.415	0.00103
**Smooth terms**	**edf**	**Ref.df**	***F***	***p***
s(Temp_num): TreatmentControl	4.577	4.917	22.728	< 2e‐16
s(Temp_num): TreatmentLow	4.356	4.815	21.678	< 2e‐16
s(Temp_num): TreatmentHigh	3.105	3.734	3.206	0.0271

## Appendix 12

Generalised Additive Model (GAM) output tables analysing the effect of clipping treatment on the leaf nutrient contents of *H. tasmanica* . Refer to Appendix 8 for detail on the statistical tests.

**Table:**

***H. tasmanica*** —**Leaf Carbon (mass %)**				
**Parametric coefficients**	**Estimate**	**Std. Error**	***t***	**Pr(>|t|)**
(Intercept)	−1.02912	0.02808	−36.649	< 2e‐16
TreatmentLow	0.28013	0.03359	8.34	< 2e‐16
TreatmentHigh	0.25031	0.03348	7.476	7.68E‐14
**Smooth terms**	**edf**	**Ref.df**	***F***	***p***
s(Temp_num): TreatmentControl	4.955	4.999	194.56	< 2e‐16
s(Temp_num): TreatmentLow	2.743	3.368	10.52	0.0229
s(Temp_num): TreatmentHigh	3.284	3.939	35.45	5.62E‐07
** *H. tasmanica* —Leaf Nitrogen (mass %)**				
**Parametric coefficients**	**Estimate**	**Std. Error**	***z***	**Pr(>|z|)**
(Intercept)	(Intercept)	1.03049	0.0287	35.901
TreatmentLow	TreatmentLow	0.07916	0.04059	1.95
TreatmentHigh	TreatmentHigh	0.04733	0.04023	1.177
**Smooth terms**	**edf**	**Ref.df**	**Chi.sq**	***p***
s(Temp_num): TreatmentControl	4.094	4.658	19.543	< 2e‐16
s(Temp_num): TreatmentLow	2.695	3.312	5.628	0.00109
s(Temp_num): TreatmentHigh	1	1	7.19	0.00911
** *H. tasmanica* —Leaf C:N (molar)**				
**Parametric coefficients**	**Estimate**	**Std. Error**	***t***	**Pr(>|t|)**
(Intercept)	28.2508	0.8348	33.841	< 2e‐16
TreatmentLow	1.5026	1.1805	1.273	0.207
TreatmentHigh	1.6349	1.1703	1.397	0.167
**Smooth terms**	**edf**	**Ref.df**	***F***	***p***
s(Temp_num): TreatmentControl	3.865	4.486	8.832	5.63E‐06
s(Temp_num): TreatmentLow	2.456	3.037	5.641	0.001522
s(Temp_num): TreatmentHigh	1.66	2.06	8.221	0.000533

## Appendix 13

Tukey's Honestly Significant Difference (HSD) posthoc test results for the effect of clipping treatment on the leaf nutrient contents of *P. australis* ; significant differences (*p* < 0.05) are highlighted in bold; CT, control treatment; LT, low clipping treatment; HT, high clipping treatment. Refer to Appendix 8 for detail on the statistical tests.

**Table:**

*P. australis* —Leaf nutrients						
	Temperature	Contrast	Estimate	SE	*t* ratio	*p*
Carbon (mass %)	6	CT–LT	1.487	0.628	2.366	0.053
	6	CT–HT	−0.340	0.519	−0.655	0.790
	6	LT–HT	−1.827	0.511	−3.553	**0.002**
	10	CT–LT	0.377	0.632	0.598	0.822
	10	CT–HT	−0.543	0.500	−1.084	0.527
	10	LT–HT	−0.921	0.498	−1.841	0.163
	16	CT–LT	0.404	0.629	0.642	0.797
	16	CT–HT	−0.973	0.484	−2.001	0.119
	16	LT–HT	−1.377	0.482	−2.840	**0.016**
	22	CT–LT	0.985	0.639	1.541	0.278
	22	CT–HT	0.571	0.493	1.162	0.479
	22	LT–HT	−0.413	0.488	−0.845	0.676
	26	CT–LT	−0.146	0.645	−0.226	0.972
	26	CT–HT	0.704	0.503	1.403	0.344
	26	LT–HT	0.850	0.504	1.692	0.215
	32	CT–LT	0.045	0.060	0.744	0.738
	32	CT–HT	−33.955	0.272	−140.725	**< 0.001**
	32	LT–HT	−34.000	0.272	−141.630	**< 0.001**
Nitrogen (mass %)	6	CT–LT	−0.002	0.091	−0.018	1.000
	6	CT–HT	0.142	0.089	1.598	0.253
	6	LT–HT	0.144	0.091	1.592	0.255
	10	CT–LT	0.143	0.081	1.766	0.188
	10	CT–HT	0.034	0.075	0.456	0.892
	10	LT–HT	−0.108	0.080	−1.358	0.368
	16	CT–LT	0.117	0.083	1.411	0.341
	16	CT–HT	−0.077	0.079	−0.973	0.596
	16	LT–HT	−0.194	0.082	−2.357	0.054
	22	CT–LT	−0.074	0.079	−0.943	0.615
	22	CT–HT	−0.026	0.074	−0.348	0.935
	22	LT–HT	0.048	0.078	0.624	0.807
	26	CT–LT	−0.167	0.080	−2.071	0.103
	26	CT–HT	−0.155	0.075	−2.061	0.105
	26	LT–HT	0.011	0.079	0.143	0.989
	32	CT–LT	0.002	0.093	0.018	1.000
	32	CT–HT	−0.772	0.092	−8.368	**< 0.001**
	32	LT–HT	−0.774	0.093	−8.322	**< 0.001**
C:N (molar)	6	CT–LT	0.051	0.104	0.492	0.875
	6	CT–HT	−0.140	0.101	−1.394	0.349
	6	LT–HT	−0.191	0.100	−1.908	0.143
	10	CT–LT	−0.141	0.099	−1.418	0.337
	10	CT–HT	−0.068	0.091	−0.753	0.733
	10	LT–HT	0.072	0.089	0.814	0.696
	16	CT–LT	−0.115	0.100	−1.148	0.488
	16	CT–HT	0.039	0.093	0.424	0.906
	16	LT–HT	0.154	0.092	1.681	0.219
	22	CT–LT	0.080	0.097	0.821	0.691
	22	CT–HT	0.053	0.089	0.596	0.822
	22	LT–HT	−0.027	0.087	−0.305	0.950
	26	CT–LT	0.209	0.099	2.123	0.092
	26	CT–HT	0.122	0.090	1.348	0.374
	26	LT–HT	−0.087	0.089	−0.984	0.589
	32	CT–LT	0.043	0.105	0.412	0.911
	32	CT–HT	−0.887	0.103	−8.612	**< 0.001**
	32	LT–HT	−0.930	0.103	−9.046	**< 0.001**

## Appendix 14

Tukey's Honestly Significant Difference (HSD) posthoc test results for the effect of clipping treatment on the leaf nutrient contents of *H. tasmanica* ; significant differences (*p* < 0.05) are highlighted in bold; CT, control treatment; LT, low clipping treatment; HT, high clipping treatment. Refer to Appendix 8 for detail on the statistical tests.

**Table:**

*H. tasmanica* —Leaf nutrients						
	Temperature	Contrast	Estimate	SE	*t* ratio	*p*
Carbon (mass %)	6	CT–LT	1.660	1.251	1.774	0.386
	6	CT–HT	4.017	1.256	6.040	**0.006**
	6	LT–HT	2.357	1.181	4.134	0.121
	10	CT–LT	−1.368	1.131	1.489	0.452
	10	CT–HT	0.614	1.161	4.208	0.858
	10	LT–HT	1.982	0.974	2.868	0.112
	16	CT–LT	0.262	1.183	0.734	0.973
	16	CT–HT	0.890	1.217	0.240	0.746
	16	LT–HT	0.628	1.056	−0.241	0.824
	22	CT–LT	0.251	1.156	−0.266	0.974
	22	CT–HT	−0.635	1.193	−2.231	0.856
	22	LT–HT	−0.886	0.994	−1.994	0.648
	26	CT–LT	0.288	1.168	−0.818	0.967
	26	CT–HT	−0.599	1.199	−0.985	0.872
	26	LT–HT	−0.887	1.024	−0.282	0.664
	32	CT–LT	−28.091	1.314	−1.376	**< 0.001**
	32	CT–HT	−27.734	1.163	3.722	**< 0.001**
	32	LT–HT	0.357	1.682	4.230	0.975
Nitrogen (mass %)	6	CT–LT	−0.060	0.088	−0.681	0.775
	6	CT–HT	0.096	0.080	1.197	0.459
	6	LT–HT	0.156	0.077	2.041	0.110
	10	CT–LT	−0.002	0.076	−0.023	1.000
	10	CT–HT	0.021	0.071	0.294	0.954
	10	LT–HT	0.023	0.060	0.374	0.926
	16	CT–LT	0.028	0.079	0.357	0.932
	16	CT–HT	−0.017	0.067	−0.253	0.965
	16	LT–HT	−0.045	0.058	−0.780	0.716
	22	CT–LT	0.035	0.075	0.462	0.889
	22	CT–HT	0.042	0.066	0.630	0.804
	22	LT–HT	0.007	0.057	0.127	0.991
	26	CT–LT	−0.058	0.077	−0.746	0.737
	26	CT–HT	−0.032	0.071	−0.452	0.894
	26	LT–HT	0.025	0.063	0.405	0.914
	32	CT–LT	−0.805	0.138	−5.839	**< 0.001**
	32	CT–HT	−0.789	0.116	−6.804	**< 0.001**
	32	LT–HT	0.016	0.108	0.150	0.988
C:N (molar)	6	CT–LT	2.047	2.53	0.808	0.699
	6	CT–HT	0.742	2.43	0.305	0.950
	6	LT–HT	−1.305	2.31	−0.564	0.840
	10	CT–LT	−0.919	2.13	−0.43	0.903
	10	CT–HT	0.072	2.04	0.035	0.999
	10	LT–HT	0.991	1.75	0.567	0.838
	16	CT–LT	−0.615	2.22	−0.277	0.959
	16	CT–HT	1.575	2.06	0.764	0.726
	16	LT–HT	2.190	1.80	1.218	0.447
	22	CT–LT	0.565	2.10	0.269	0.961
	22	CT–HT	0.872	1.98	0.441	0.899
	22	LT–HT	0.307	1.72	0.178	0.983
	26	CT–LT	−1.138	2.18	−0.522	0.861
	26	CT–HT	−2.379	2.07	−1.151	0.486
	26	LT–HT	−1.241	1.83	−0.678	0.777
	32	CT–LT	−17.475	3.93	−4.441	**< 0.001**
	32	CT–HT	−21.042	3.53	−5.955	**< 0.001**
	32	LT–HT	−3.568	3.26	−1.093	0.521

## Appendix 15

Total chlorophyll content (Chltot; mg gDW^−1^) and Chl*a*/Chl*b* for *P. australis* (A) and *H. tasmanica* (B) across temperature treatments (6°C–32°C); Bars show mean values +/− standard deviation (SD; errorbars) and are in the order of control treatment (Control; green), low clipping treatment (Low; orange) and high clipping treatment (High; purple) for each temperature.

## Appendix 16

Generalised Additive Model (GAM) output tables analysing the effect of clipping treatment on the pigment contents of *P. australis* . Refer to Appendix 8 for detail on the statistical tests.

**Table:**

***P. australis***					
	**Parametric coefficients**	**Estimate**	**Std. Error**	***t***	**Pr(>|t|)**
Chl *a* (mg gDW^−1^)	(Intercept)	0.0705	0.08909	0.791	0.431
	TreatmentLow	0.12092	0.12599	0.96	0.3399
	TreatmentHigh	−0.21453	0.12599	−1.703	0.0923
	**Smooth terms**	**edf**	**Ref.df**	***F***	***p***
	s(Temp_num)	3.448	4.089	2.642	0.0367
Chl *b* (mg gDW^−1^)	**Parametric coefficients**	**Estimate**	**Std. Error**	***z***	**Pr(>|z|)**
	(Intercept)	−0.53799	0.08987	−5.986	5.18E‐08
	TreatmentLow	0.11327	0.12709	0.891	0.3753
	TreatmentHigh	−0.23097	0.12709	−1.817	0.0728
	**Smooth terms**	**edf**	**Ref.df**	**Chi.sq**	***p***
	s(Temp_num)	3.478	4.12	3.002	0.021
Chl*a*/Chl*b*	**Parametric coefficients**	**Estimate**	**Std. Error**	***t***	**Pr(>|t|)**
	(Intercept)	0.608485	0.007417	82.039	< 2e‐16
	TreatmentLow	0.007646	0.010489	0.729	0.468
	TreatmentHigh	0.016441	0.010489	1.567	0.121
	**Smooth terms**	**edf**	**Ref.df**	***F***	***p***
	s(Temp_num)	2.841	3.443	15.62	< 2e‐16
Chl *total* (mg gDW^−1^)	**Parametric coefficients**	**Estimate**	**Std. Error**	***t***	**Pr(>|t|)**
	(Intercept)	0.50528	0.08928	5.66	2.08E‐07
	TreatmentLow	0.11817	0.12626	0.936	0.352
	TreatmentHigh	−0.22022	0.12626	−1.744	0.0848
	**Smooth terms**	**edf**	**Ref.df**	***F***	***p***
	s(Temp_num)	3.459	4.101	2.752	0.031

## Appendix 17

Generalised Additive Model (GAM) output tables analysing the effect of clipping treatment on the pigment contents of *H. tasmanica* . Refer to Appendix 8 for detail on the statistical tests.

**Table:**

*H. tasmanica*					
	Parametric coefficients	Estimate	Std. Error	*t* value	Pr(>|t|)
Chl *a* (mg gDW^−1^)	(Intercept)	−0.21886	0.08168	−2.679	0.00906
	TreatmentLow	−0.12473	0.11667	−1.069	0.28846
	TreatmentHigh	0.03176	0.1166	0.272	0.78609
	**Smooth terms**	**edf**	**Ref.df**	***F***	***p***
	s(Temp_num)	3.979	4.57	4.175	0.00187
Chl *b* (mg gDW^−1^)	**Parametric coefficients**	**Estimate**	**Std. Error**	***z***	**Pr(>|z|)**
	(Intercept)	−0.76882	0.06056	−12.695	< 2e‐16
	TreatmentLow	−0.10497	0.0865	−1.214	0.229
	TreatmentHigh	0.019	0.08645	0.22	0.827
	**Smooth terms**	**edf**	**Ref.df**	**Chi.sq**	***p***
	s(Temp_num)	3.885	4.495	4.061	0.00239
Chl*a*/Chl*b*	**Parametric coefficients**	**Estimate**	**Std. Error**	***t***	**Pr(>|t|)**
	(Intercept)	0.505402	0.003321	152.161	< 2e‐16
	TreatmentLow	0.001519	0.004744	0.32	0.749
	TreatmentHigh	−0.003374	0.004741	−0.712	0.477
	**Smooth terms**	**edf**	**Ref.df**	***F***	***p***
	s(Temp_num)	4.179	4.711	33.31	9.80E‐06
Chl *total* (mg gDW^−1^)	**Parametric coefficients**	**Estimate**	**Std. Error**	***t***	**Pr(>|t|)**
	(Intercept)	0.18183	0.0979	1.857	0.0672
	TreatmentLow	−0.15639	0.13984	−1.118	0.267
	TreatmentHigh	0.03587	0.13975	0.257	0.7981
	**Smooth terms**	**edf**	**Ref.df**	***F***	***p***
	s(Temp_num)	3.954	4.55	4.113	0.00209
